# Proteome-wide analyses reveal diverse functions of protein acetylation and succinylation modifications in fast growing stolons of bermudagrass (*Cynodon dactylon* L.)

**DOI:** 10.1186/s12870-022-03885-2

**Published:** 2022-10-27

**Authors:** Bing Zhang, Zhuoting Chen, Qixue Sun, Jianxiu Liu

**Affiliations:** 1 College of Animal Science and Technology, Yangzhou University, Yangzhou, 225009 China; 2 Institute of Botany, Jiangsu Province and Chinese Academy of Sciences, Nanjing, 210014 China

**Keywords:** Bermudagrass, *Cynodon dactylon*, Stolon, Metabolism, Acetylation, Succinylation

## Abstract

**Background:**

Bermudagrass (*Cynodon dactylon* L.) is an important warm-season turfgrass species with well-developed stolons, which lay the foundation for the fast propagation of bermudagrass plants through asexual clonal growth. However, the growth and development of bermudagrass stolons are still poorly understood at the molecular level.

**Results:**

In this study, we comprehensively analyzed the acetylation and succinylation modifications of proteins in fast-growing stolons of the bermudagrass cultivar Yangjiang. A total of 4657 lysine acetylation sites on 1914 proteins and 226 lysine succinylation sites on 128 proteins were successfully identified using liquid chromatography coupled to tandem mass spectrometry, respectively. Furthermore, 78 proteins and 81 lysine sites were found to be both acetylated and succinylated. Functional enrichment analysis revealed that acetylated proteins regulate diverse reactions of carbohydrate metabolism and protein turnover, whereas succinylated proteins mainly regulate the citrate cycle. These results partly explained the different growth disturbances of bermudagrass stolons under treatment with sodium butyrate and sodium malonate, which interfere with protein acetylation and succinylation, respectively. Moreover, 140 acetylated proteins and 42 succinylated proteins were further characterized having similarly modified orthologs in other grass species. Site-specific mutations combined with enzymatic activity assays indicated that the conserved acetylation of catalase and succinylation of malate dehydrogenase both inhibited their activities, further implying important regulatory roles of the two modifications.

**Conclusion:**

In summary, our study implied that lysine acetylation and succinylation of proteins possibly play important regulatory roles in the fast growth of bermudagrass stolons. The results not only provide new insights into clonal growth of bermudagrass but also offer a rich resource for functional analyses of protein lysine acetylation and succinylation in plants.

**Supplementary Information:**

The online version contains supplementary material available at 10.1186/s12870-022-03885-2.

## Background

Through the addition of different modifying groups to specific amino acids, protein post-translational modifications (PTMs) fine-tune the function of proteins by altering their activity, turnover, localization, and interaction partners [1]. To data, hundreds of PTMs have been characterized in diverse organisms, among which disulfide bond formation at cysteine, phosphorylation at serine/threonine/tyrosine, ubiquitination at lysine, methylation at lysine and arginine, N-glycosylation at asparagine, and O-glycosylation at serine/threonine/tyrosine, are the most commonly observed PTMs [1]. In addition to these PTMs, lysine acetylation and succinylation are two other PTMs with important regulatory functions. Reversible lysine acetylation was firstly identified in histone proteins isolated from the calf thymus approximately 60 years ago and found to play crucial roles in eukaryote gene expression regulation [2]. Since then, many studies have revealed that lysine acetylation is actually a widespread PTM existing in both histone and non-histone proteins [3]. By contrast, lysine succinylation was firstly identified in isocitrate dehydrogenase from *Escherichia coli* only 10 years ago [4]. Subsequent studies also found that lysine succinylation is a frequently occurring PTM and overlaps extensively with acetylation [5].

In recent years, advances in high-resolution mass-spectrometry technology and the availability of modification-specific antibodies have facilitated the global identification of protein lysine acetylation and succinylation in plants. For example, two pioneer studies only identified 64 and 91 lysine acetylation sites on 57 and 74 proteins in *Arabidopsis*, respectively [6, 7]. In an updated acetylome profiling of *Arabidopsis* plants, the number of acetylation sites and acetylated proteins increased to 2152 and 1022, respectively [8]. Similarly, an early study only identified 60 lysine acetylation sites on 44 proteins in rice [9]; however, the latest study successfully identified 1669 lysine acetylation sites on 1024 rice proteins [10]. Meanwhile, the numbers of succinylation sites and succinylated proteins identified in rice also increased from 665 and 261 to 5502 and 2593, respectively [10, 11]. Acetylome and succinylome analyses were also conducted in other plant species, including wheat, maize, soybean, tomato, patchouli, strawberry, paper mulberry, *Ananas comosus*, *Camellia sinensis*, *Carya cathayensis*, *Catalpa bungei*, *Chlamydomonas reinhardtii*, *Dendrobium officinale*, *Eucommia ulmoides* Oliver, *Kandelia candel*, *Picea asperata*, *Phaeodactylum tricornutum*, and *Physcomitrium patens* [12–32]. Moreover, the interplay between acetylation and succinylation was also analyzed through simultaneous identification and comparison of the two PTMs in rice and *Brachypodium distachyon* [11, 33]. The results of these studies collectively revealed the in-depth participation of the two PTMs in the regulation of plant growth, development and stress responses.

As a perennial warm-season turfgrass species with great economic values, bermudagrass (*Cynodon dactylon* L.) is widely used for turf planting in public parks, golf courses, sport fields and home lawns in warm areas around the world [34]. Unlike domesticated cereal grasses such as rice, wheat, maize, sorghum and barley, bermudagrass plants usually reproduce asexually through clonal regeneration of seedlings from stolon nodes [35]. With this reproductive characteristic, bermudagrass can quickly spread in an open location by constantly generating and regenerating stolons, laying the foundation for turf-type cultivars of bermudagrass to form uniform and beautiful turfs. In the past several years, high-throughput transcriptomics and proteomics studies have revealed the pivotal role of metabolic regulation in the specialization and prostrate growth of bermudagrass stolons. For example, comparative transcriptome analysis indicated that lignin biosynthesis was compromised in stolons of prostrate-growing wild germplasm in comparison with erect-growing wild germplasm [36]. Comparative proteomics and enzymatic analyses demonstrated that starch accumulation was highly active in stolons, whereas glycolysis activity was more prominent in shoots [37]. Compared with underground-growing rhizomes, aboveground-growing stolons have a substantial amount of chlorophyll and high expression of enzymes involved in photosynthesis, suggesting that the stolon might function as an extra photosynthetic organ [38]. However, the detailed molecular mechanisms regulating the different metabolic reactions in bermudagrass stolons remain poorly understood.

In the current study, we performed the global identification of protein lysine acetylation and succinylation modifications in fast growing bermudagrass stolons. A total of 4657 lysine acetylation sites on 1914 proteins and 226 lysine succinylation sites on 128 proteins were successfully identified. The features and functions of the two PTMs in fast growing bermudagrass stolons were systematically analyzed, whereas the influences of the two PTMs on the enzymatic activities of key enzymes were also evaluated. The results of this study not only provide the first comprehensive view of the acetylome and succinylome in turfgrasses but also expand the understanding of metabolic regulation in fast growing plant organs.

## Results

### Salts interfering with protein lysine acetylation and succinylation could modulate the growth of bermudagrass stolons

**Fig. 1:**
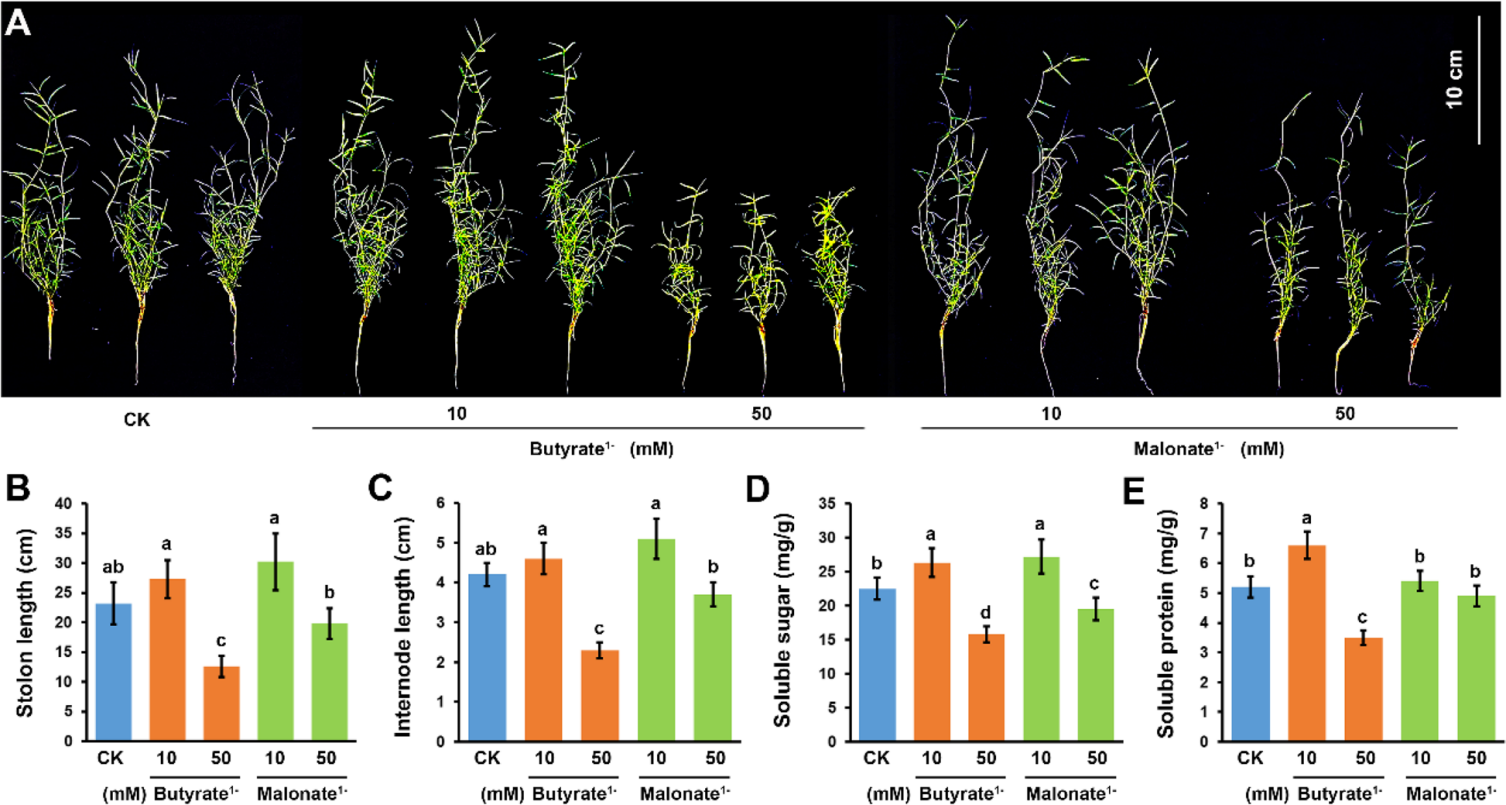
Phenotypes of bermudagrass cultivar Yangjiang under sodium butyrate and sodium malonate treatments (**A**) Plant morphology, (**B**) stolon length, (**C**) stolon internode length, (**D**) soluble sugar content, and (**E**) soluble protein content of the bermudagrass cultivar Yangjiang growing in Hoagland's nutrient solution without (CK) and with 10/50 mM sodium butyrate or sodium malonate treatments for two weeks. Error bars represent the SE of three biological replicates. Different letters indicate significant differences determined by Tukey’s multiple comparison test

Stolons of bermudagrass cultivar Yangjiang grow very fast under normal growth conditions. In two weeks, the length of newly generated stolons reached 23.2 cm on average, whereas the stolon internode length was approximately 4.2 cm (Fig. 1A-C). The addition of 10 mM sodium butyrate, an inhibitor of histone deacetylase [39], slightly promoted the growth of stolons (Fig. 1A-C). However, the growth of stolons was severely inhibited under 50 mM sodium butyrate treatment that the stolon length and stolon internode length were significantly shortened to 12.6 cm and 2.3 cm, respectively (Fig. 1B and C). The addition of 10 mM sodium malonate, an inhibitor of succinate dehydrogenase [40], also slightly promoted the growth of stolons (Fig. 1A-C). By contrast, increasing the sodium malonate concentration to 50 mM resulted in the inhibition of stolon growth since the stolon length and stolon internode length were shortened to 19.8 cm and 3.7 cm, respectively (Fig. 1B and C).

The contents of soluble sugar and protein are important physiological indicators in plant growth and development [41, 42]. Stolons of bermudagrass cultivar Yangjiang accumulate substantial amounts of soluble sugar and protein (22.5 mg/g and 5.2 mg/g, respectively) under normal growth conditions (Fig. 1D and E). Low concentration (10 mM) treatment of sodium butyrate and sodium malonate both significantly increased the soluble sugar content, whereas high concentration (50 mM) of the two salts both significantly inhibited the accumulation of soluble sugar (Fig. 1D). Similarly, the addition of 10 mM and 50 mM sodium butyrate increased and decreased the protein content in bermudagrass stolons, respectively (Fig. 1E). However, the soluble protein content remained unchanged under different sodium malonate treatments (Fig. 1E). In conjunction with the western blot detection results that protein acetylation and succinylation levels were decreased under sodium butyrate and sodium malonate treatments, respectively (Fig. S1); the above observations collectively implied that protein lysine acetylation and succinylation might play important roles in the growth of bermudagrass stolons by modulating the soluble sugar and/or protein contents.

### Identification of lysine acetylated and succinylated proteins in bermudagrass stolons

**Fig. 2:**
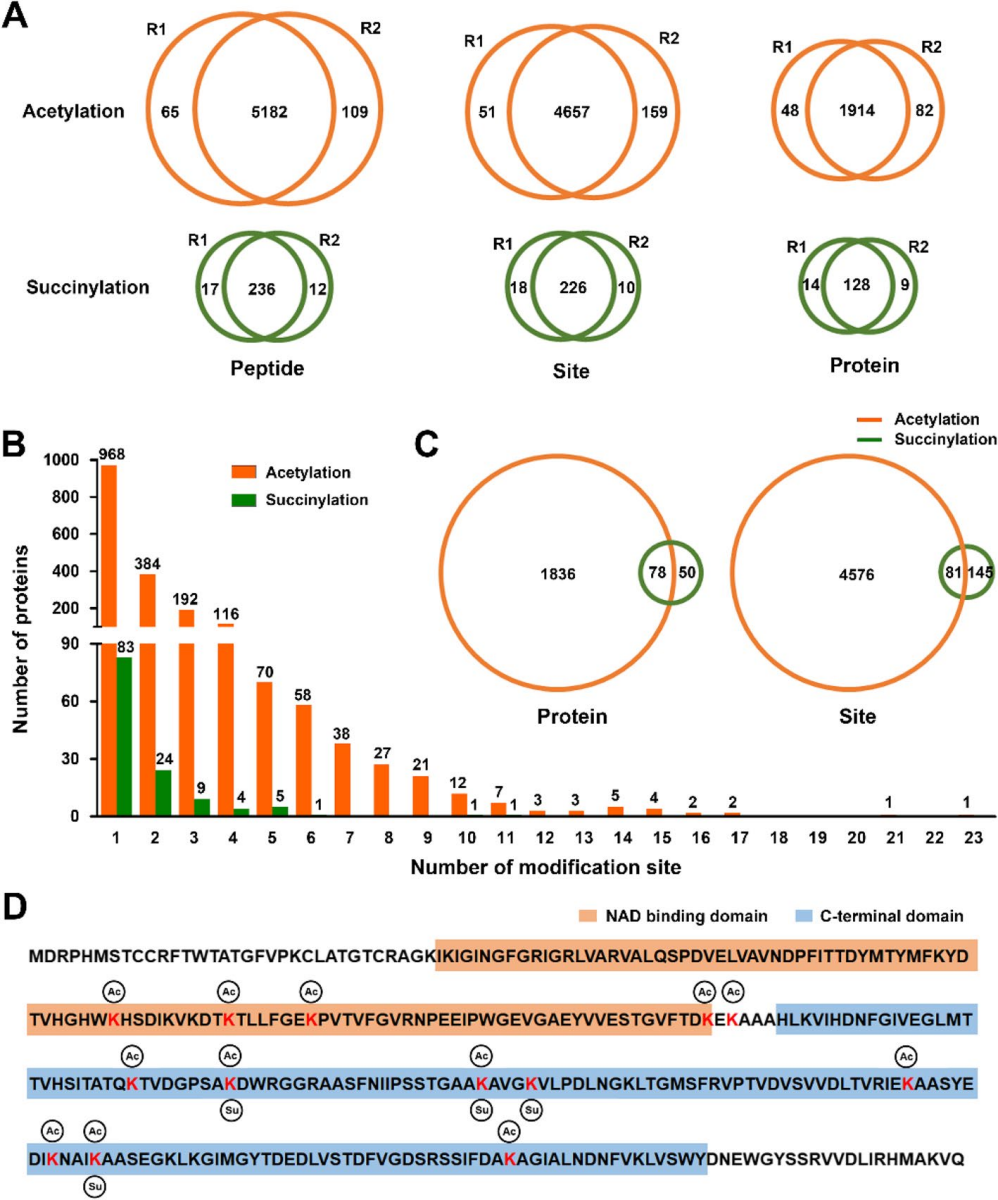
Overview of acetylated and succinylated sites, peptides and proteins in bermudagrass stolons (**A**) Number of acetylated/succinylated peptides, acetylation/succinylation sites, and acetylated/succinylated proteins identified by LC–MS/MS. (**B**) Distribution of acetylation and succinylation sites in different proteins. (**C**) Overlap between acetylated and succinylated proteins/sites. (**D**) Identified acetylation and succinylation sites on cytosolic glyceraldehyde-3-phosphate dehydrogenase protein (genome accession number: Cd5B2G000610.3)

To obtain the whole acetylome and succinylome profiles and determine the detailed functions of acetylation and succinylation modifications (affected and unaffected by sodium butyrate and sodium malonate) in growing bermudagrass stolons, proteome-wide acetylation and succinylation analyses were performed through antibody affinity enrichment and liquid chromatography coupled to tandem mass spectrometry (LC–MS/MS). A total of 241,999 spectra were finally obtained and 40.78% of them were matched to the bermudagrass genome with a < 0.02 Da of precursor mass tolerance (Fig. S2; Table S1). Among these matched peptides, 5182 acetylated peptides and 236 succinylated peptides were reproducibly identified in two replicates (Fig. 2A). The average percentage overlap between the two replicates of identified acetylated and succinlylated peptides was 98.35% and 94.22%, respectively. Based on these identifications, 4657 nonredundant acetylation sites and 226 nonredundant succinylation sites were successfully mapped to 1914 and 128 proteins, respectively (Fig. 2A; Table S2 and S3).

The number of acetylation sites varied greatly in different acetylated proteins. Specifically, 968 proteins had only one acetylation site, whereas 384, 192, 116, 70, and 58 proteins had two, three, four, five, and six acetylation sites, respectively (Fig. 2B). Notably, two proteins, elongation factor 2 (genome accession number: Cd2B1G011420.1) and 5-methyltetrahydropteroyltriglutamate--homocysteine methyltransferase 1 (Cd2A1G017400.1), were found to have more than 20 acetylation sites (Table S2). Although the number of identified succinylation sites was much lower than that of acetylation sites, a similarly uneven distribution of succinylation sites was also observed. Specifically, 83 proteins had only one succinylation site, whereas 24, 9, 4, and 5 proteins had two, three, four, and five succinylation sites, respectively (Fig. 2B). Two proteins, dihydrolipoyl dehydrogenase (Cd2B2G019840.1) and mitochondrial ATP synthase subunit delta (Cd5A2G009880.1), were found to have more than 10 succinylation sites (Table S3). Interestingly, 78 proteins were found to be both acetylated and succinylated, whereas 81 lysine sites of 56 proteins were simultaneously acetylated and succinylated (Fig. 2C; Table S4). For example, 12 acetylation sites and 4 succinylation sites were identified in a cytosolic glyceraldehyde-3-phosphate dehydrogenase protein (Cd5B2G000610.3), and three sites were found to be simultaneously acetylated and succinylated (Fig. 2D; Table S4).

**Fig. 3:**
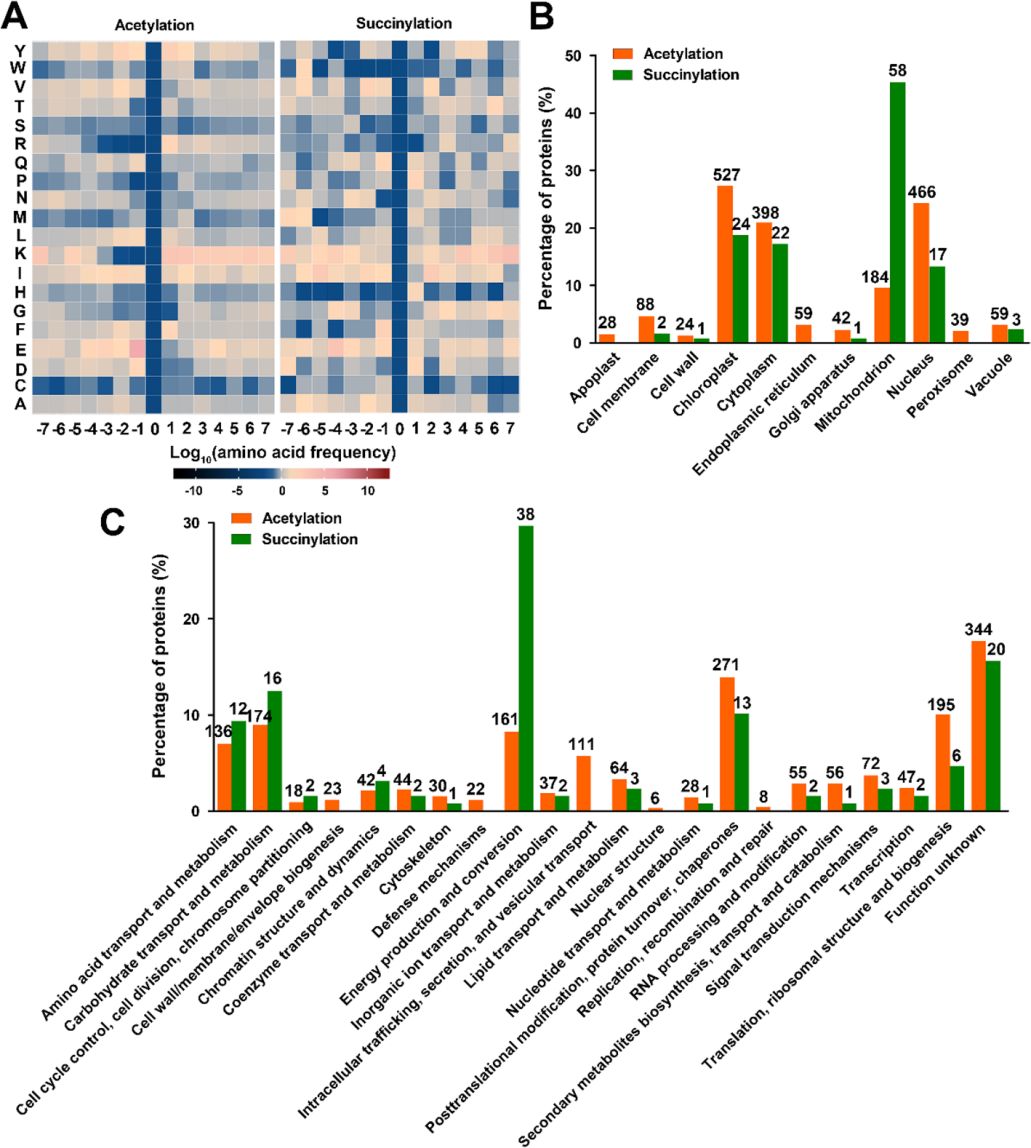
Sequence and functional diversity of acetylated and succinylated proteins in bermudagrass stolons (**A**) Heatmap showing the relative abundances of different amino acids flanking the acetylation and succinylation sites. (**B**) Subcellular distribution of acetylated and succinylated proteins. (**C**) COG functional classification of the acetylated and succinylated proteins

To better understand the features of acetylation and succinylation sites, the occurrence frequencies of different amino acids from positions -7 to + 7 flanking the modified lysine sites were analyzed and compared. The results indicated that the frequencies of tyrosine (Y) and glutamic acid (E) were higher at -4 to -1 upstream positions around the acetylated lysine site, whereas the frequency of lysine was higher in all downstream positions (+ 1 to + 7) as well as -7 and -5 upstream positions (Fig. 3A). The frequency of lysine was also higher at -7 to -4 upstream positions and + 4 to + 7 downstream positions around the succinylated lysine site, whereas isoleucine (I), phenylalanine (F) and glutamic acid were more frequently distribute at -5, -1 and -3 upstream positions around the succinylated lysine site (Fig. 3A). Moreover, 14 conserved acetylation motifs and two conserved succinylation motifs were successfully identified by Motif-X (Fig. S3). The results also indicated that glutamic acid and lysine residues are significantly enriched around the acetylation site, whereas phenylalanine is significantly enriched at the -1 upstream position around the succinylation site.

The subcellular localizations of acetylated and succinylated proteins were also analyzed. The results indicated that acetylated proteins were mainly located in the chloroplast, nucleus, cytoplasm and mitochondrion, which accounted for 27.53%, 24.35%, 20.79% and 9.61% of the identified acetylated proteins, respectively (Fig. 3B). By contrast, nearly half of the succinylated proteins (58/128) were located in the mitochondrion, and 24, 22 and 17 succinylated proteins were located in chloroplast, cytoplasm and nucleus, respectively (Fig. 3B). In line with the subcellular location analysis results, functional annotation indicated that 271, 195, 174, 161 and 136 acetylated proteins were classified as involved in posttranslational modification, protein turnover, chaperones; translation, ribosomal structure and biogenesis; carbohydrate transport and metabolism; energy production and conversion; and amino acid transport and metabolism, respectively, whereas 38 of the 128 succinylated proteins were categorized into energy production and conversion (Fig. 3C). These results collectively illustrated that acetylated and succinylated proteins are distributed unevenly in different subcellular locations to execute diverse functions in bermudagrass stolons.

### Biased protein lysine acetylation and succinylation in different metabolic pathways

**Fig. 4:**
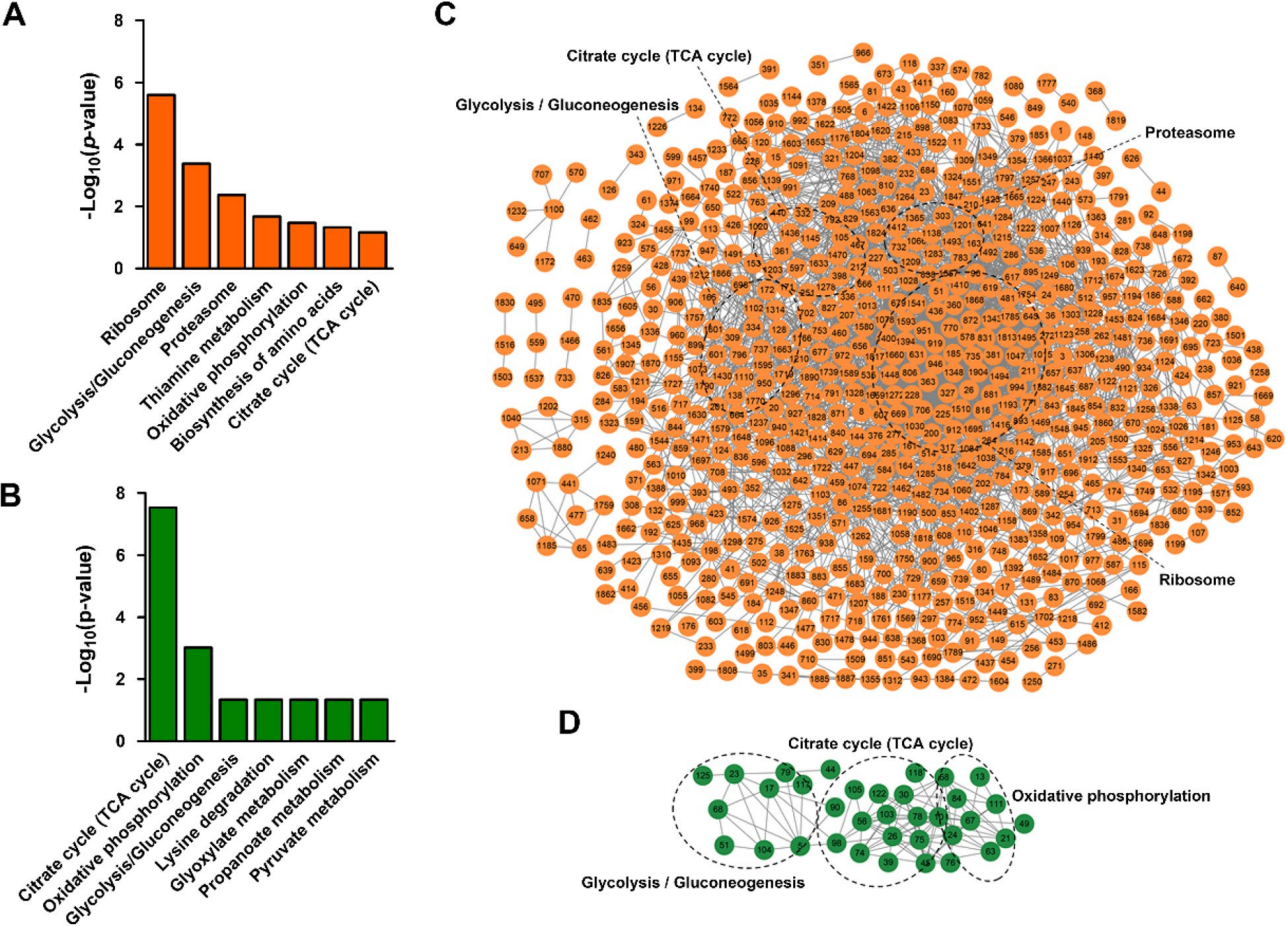
Functional enrichment and protein–protein interaction analyses of acetylated and succinylated proteins in bermudagrass stolons. Biochemical pathways significantly enriched with (**A**) acetylated proteins, and (**B**) succinylated proteins. Protein–protein interaction network of (**C**) acetylated proteins, and (**D**) succinylated proteins showing the enriched biochemical pathways

The functional classification results implied that acetylated and succinylated proteins are involved in different cellular activities. To further explore their functions, Kyoto Encyclopedia of Genes and Genomes (KEGG) enrichment analyses were firstly conducted. The results indicated that acetylated and succinylated proteins were both enriched in seven biochemical pathways (Fig. 4A and B; Table S5 and S6). Specifically, ribosome and glycolysis/gluconeogenesis were the two most significantly enriched pathways for acetylated proteins, whereas the citrate cycle was the most significantly enriched pathway for succinylated proteins (Fig. 4A and B). Protein–protein interaction (PPI) analyses further indicated that 36.31% (695/1914) of acetylated proteins and 27.34% (35/128) of succinylated proteins existed in complex PPI networks, respectively (Fig. 4C and D). Interestingly, 48 ribosomal proteins and 18 enzymes involved in glycolysis/gluconeogenesis were clustered in the PPI network of acetylated proteins, whereas 15 enzymes involved in the citrate cycle (TCA cycle) were clustered in the PPI network of succinylated proteins (Fig. 4C and D).

**Fig. 5:**
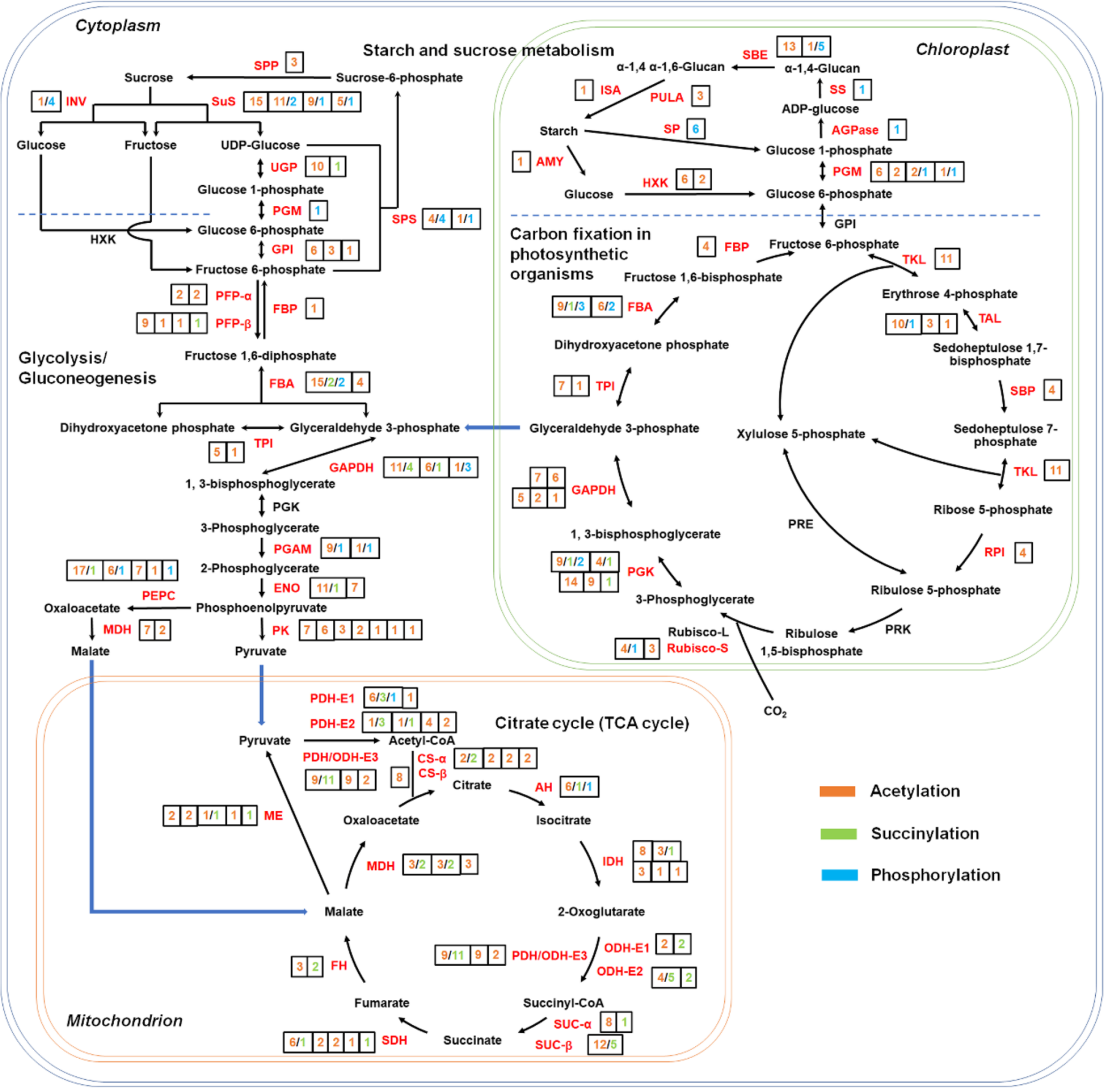
PTM-regulated carbohydrate metabolism network in bermudagrass stolons. Enzymes with/without PTMs are shown in red/black colors. The numbers of different PTMs on each enzyme are marked with different colors as indicated

In agreement with the KEGG enrichment and PPI network analysis results, 51 ribosomal large subunit proteins and 33 ribosomal small subunit proteins were identified as acetylated proteins (Fig. S4). Similarly, 27 enzymes catalyzing different reactions of glycolysis/gluconeogenesis are acetylated proteins whereas only six enzymes are succinylated proteins (Fig. 5). Specifically, all seven isoforms of pyruvate kinase (PK), which catalyzes the final rate-limiting step of glycolysis, are only modified by acetylation (Fig. 5; Table S2). By contrast, 32 proteins involved in the citrate cycle were identified as acetylated proteins, among which 12 are also modified by succinylation (Fig. 5). Interestingly, three enzymes catalyzing the generation and transformation of succinyl-CoA, including dihydrolipoyllysine-residue succinyltransferase, dihydrolipoyl dehydrogenase and succinate--CoA ligase beta subunit, are all highly succinylated at multiple lysine sites (Fig. 5; Table S3). Moreover, 20 enzymes involved in starch and sucrose metabolism were also found to be acetylated proteins; however, some key rate-limiting enzymes, including invertase and 1,4-alpha-glucan-branching enzyme, are highly phosphorylated at the same time. Other enzymes, including ADP-glucose pyrophosphorylase (AGPase), starch synthase and starch phosphorylase, are solely modified by phosphorylation (Fig. 5; Table S7). These results strongly implied that different carbohydrate metabolism pathways are preferentially regulated by acetylation, succinylation and phosphorylation modifications in bermudagrass stolons.

### Functional conservation of acetylated and succinylated proteins in fast growing organs of different grasses

**Fig. 6:**
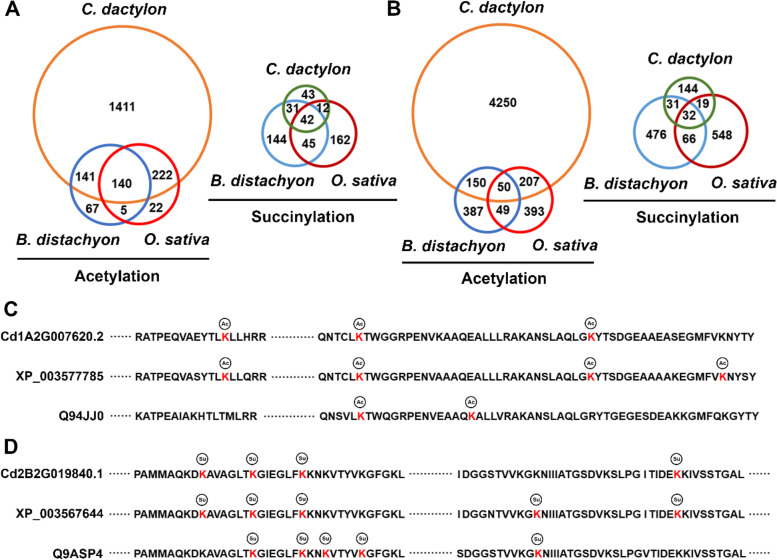
Conservation of acetylated and succinylated proteins and sites in different grass species. Venn diagram of the (**A**) acetylated and succinylated proteins, and (**B**) acetylation and succinylation sites in bermudagrass, rice and *Brachypodium distachyon*. (**C**) Identified acetylation sites on chloroplastic fructose-bisphosphate aldolase protein in bermudagrass (Cd1A2G007620.2), rice (Q94JJ0) and *B. distachyon* (XP_003577785). (**D**) Identified succinylation sites on dihydrolipoyl dehydrogenase protein in bermudagrass (Cd2B2G019840.1), rice (Q9ASP4) and *B. distachyon* (XP_003567644)

Previous studies have identified hundreds of acetylated and succinylated proteins in growing seedling leaves of *B. distachyon* and germinating seeds of rice [11, 33]. BLAST searches against these identities indicated that 362 and 281 acetylated proteins, 54 and 73 succinylated proteins of bermudagrass have acetylated and succinylated orthologs in rice and *B. distachyon*, respectively (Fig. 6A; Table S8 and S9). Specifically, 140 acetylated proteins and 42 succinylated proteins had the same modified orthologs in all three grasses. However, many of these proteins were modified at different lysine sites, thus only 50 conserved acetylation sites of 38 acetylated proteins and 32 conserved succinylation sites of 23 succinylated proteins were identified in all three grasses, respectively (Fig. 6B; Table S8 and S9). For example, three chloroplastic fructose-bisphosphate aldolase protein orthologs of bermudagrass, rice and *B. distachyon* were found to have three, four and two acetylation sites, respectively; however, sequence alignment indicated that only one acetylation site is conserved in the three species (Fig. 6C). Similarly, four, five and five lysine succinylation sites were identified in three dihydrolipoyl dehydrogenase protein orthologs of bermudagrass, rice and *B. distachyon*, respectively, however, only two succinylation sites were conserved in all three grasses (Fig. 6D).

The conserved acetylated proteins are mainly involved in translation, ribosomal structure and biogenesis; carbohydrate transport and metabolism; posttranslational modification, protein turnover, chaperones; chromatin structure and dynamics; and energy production and conversion, whereas the conserved succinylated proteins primarily participate in carbohydrate transport and metabolism; and energy production and conversion (Fig. S5). Four histone proteins, including two histone H2A (Cd4A1G007030.1 and Cd4A1G023750.1), one histone H2B (Cd2A2G026820.1) and one histone 4 (Cd7A1G012090.1), are known to participate in gene expression regulation in plant growth and development (Fig. S6; Table S10) [43]. Acetylation of the four histone proteins at conserved lysine sites might be important for fast organ growth in the three grasses; however, the detailed regulatory functions of other conserved acetylated and succinylated proteins are unclear.

**Fig. 7:**
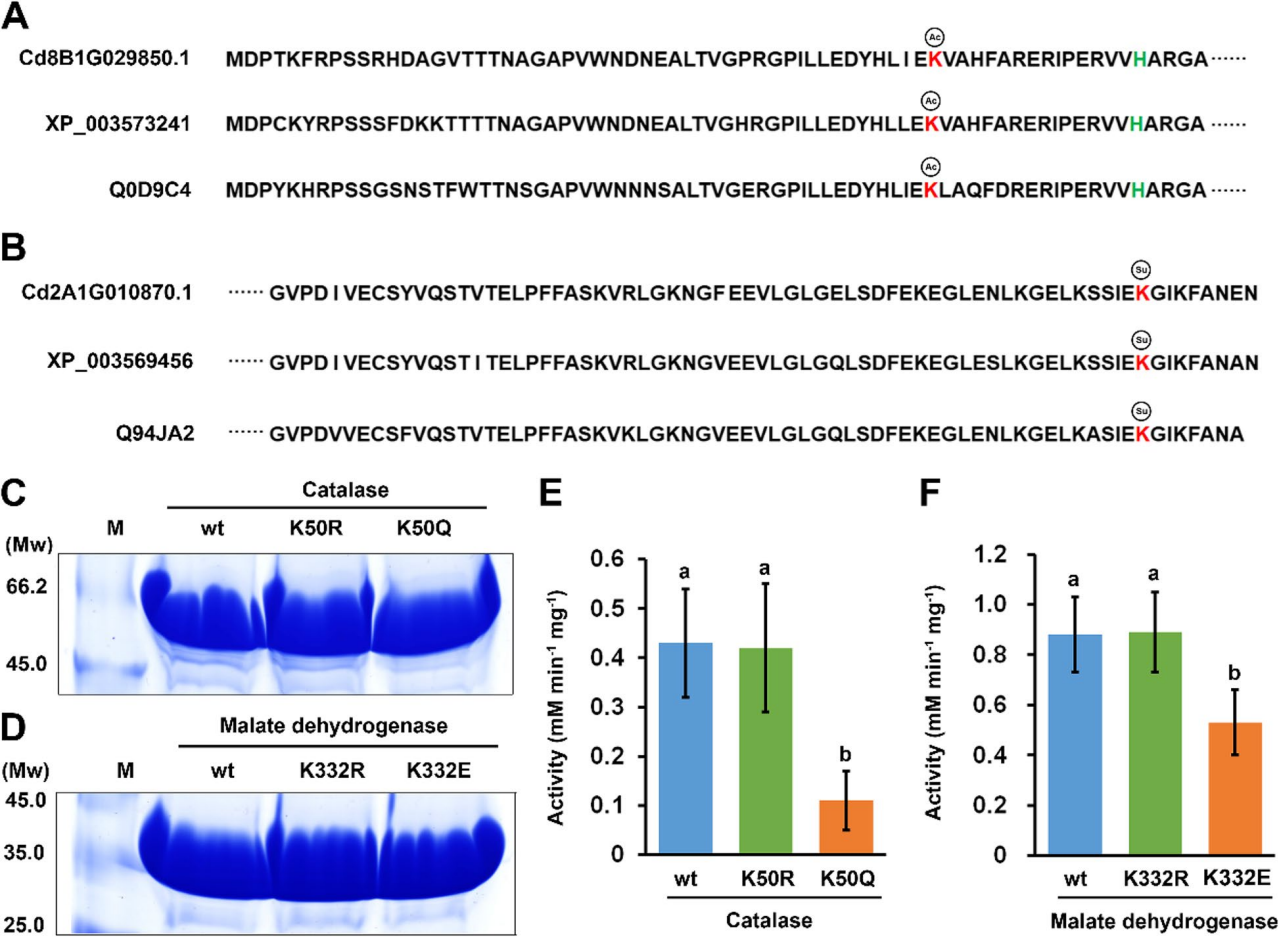
Lysine acetylation and succinylation regulate the activity of key enzymes. (**A**) Conserved acetylation modification of catalase in bermudagrass (Cd8B1G029850.1), rice (Q0D9C4) and *B. distachyon* (XP_003573241). The histidine activity site is marked in green. (**B**) Conserved succinylation modification of malate dehydrogenase in bermudagrass (Cd2A1G010870.1), rice (Q94JA2) and *B. distachyon* (XP_003569456). SDS-PAGE showing the purified His-tagged (**C**) catalase and (**D**) malate dehydrogenase proteins. Enzyme activity of the purified His-tagged (**E**) wild-type (wt), K50R and K50Q mutant catalase proteins; and (**F**) wt, K332R and K332E mutant malate dehydrogenase proteins. Error bars represent the SE of five technical replicates. Different letters indicate significant differences determined by Tukey’s multiple comparison test

Through site-specific mutation, we generated acetylation/deacetylation-mimic mutants for the acetylated catalase (CAT) protein (Cd8B1G029850.1) and succinylation/desuccinylation-mimic mutants for the succinylated malate dehydrogenase (MDH) protein (Cd2A1G010870.1), respectively (Fig. 7A-D). Enzymatic analyses revealed that the acetylation-mimic K50Q mutant CAT protein only had one quarter of the activity of the wild-type CAT protein, whereas the activity of the succinylation-mimic K332E mutant MDH protein was decreased to approximately 60% of the wild-type MDH protein (Fig. 7E, 7F). By contrast, the activities of the deacetylation-mimic K50R mutant CAT protein and desuccinylation-mimic K332R mutant MDH protein were similar to those of the wild-type CAT and MDH proteins, respectively (Fig. 7E, 7F). These results suggested that conserved acetylation and succinylation at specific lysine sites could regulate the activity of key enzymes, which could further fine-tune the fast growth of multiple organs in different grasses, especially the fast growth of stolons in bermudagrass.

## Discussion

Bermudagrass is a warm-season turfgrass species with multiple applications [44]. Through fast growth and constant differentiation of stolons, bermudagrass plants can propagate asexually in a clonal growth mode. In the past several years, many exogenic small molecules have been found to affect the growth and development of bermudagrass plants under normal and stressed conditions. For example, the addition of 10% sucrose into the growth medium promoted the prostrate growth of bermudagrass stolons [45]. Exogenous application of CaCl_2_ enhanced the freezing stress tolerance of bermudagrass seedlings, whereas pretreatment with exogenous polyamine conferred increased salt and drought tolerances of bermudagrass plants [46, 47]. Melatonin was also found to alleviate H_2_O_2_-modulated plant growth, cell damage, and reactive oxygen species accumulation in bermudagrass [48]. Additionally, the protein phosphatase inhibitor NaF and the protein kinase inhibitor staurosporine both inhibited normal growth and starch accumulation in bermudagrass stolons [49]. In this study, sodium butyrate, an inhibitor of histone deacetylase, and sodium malonate, an inhibitor of succinate dehydrogenase, were also observed to modulate the growth of bermudagrass plants, especially the stolons (Fig. 1). Interestingly, both salts promoted the growth of stolons at low concentrations (10 mM) but inhibited stolon growth at high concentrations (50 mM), which is in line with the toxic effect of high concentrations of malonic acid on the growth of *Arabidopsis* seedlings [50]. Moreover, sodium butyrate influenced the soluble sugar and protein content simultaneously, whereas sodium malonate had little effect on the soluble protein content (Fig. 1). Because bermudagrass cultivar Yangjiang is a salt-tolerance cultivar which maintains normal growth under 50 mM NaCl treatment [51], these results suggested that sodium butyrate and sodium malonate function differently to modulate the growth of bermudagrass stolons, probably by regulating the acetylation and succinylation modifications of essential proteins, respectively.

Acetylome and succinylome profiling experiments have been performed in diverse plant species. Among these studies, as many as 3179 acetylated proteins were identified in paper mulberry [20], whereas the maximum and minimum number of succinylated proteins was reported to be 2593 and 116 in rice and strawberry, respectively [10, 19]. In this study, 1914 acetylated proteins and 128 succinylated proteins were successfully identified in fast growing bermudagrass stolons (Fig. 2–4). Notably, the number of succinylated proteins was much lower than that of acetylated proteins. Although immunoaffinity enrichment efficiency might be different for the two PTMs, thus resulting in the difference between the two identification results, inherently uneven distribution of the two PTMs in bermudagrass stolons could not be excluded considering that the number of succinylated proteins identified in paper mulberry (935) was also much lower than that of acetylated proteins [20, 21]. The average acetylation and succinylation sites per protein in bermudagrass stolons were 2.43 (4657/1914) and 1.77 (226/128), respectively. These numbers are comparable with the results in other plant species. For example, the average acetylation sites per protein in soybean leaves was 2.05 (3148/1538) [16], whereas the average succinylation sites per protein was 1.79 (653/365) in *E. ulmoides* Oliver [28]. Notably, two proteins were identified to have more than 10 succinylation sites in bermudagrass stolons (Fig. 2). This result further supported the suspicion that protein lysine acetylation and succinylation might exist in bermudagrass stolons at different frequencies because multiple succinylated peptides of a single protein are rarely obtained from inefficient immunoaffinity enrichment processes. Additionally, amino acid composition analysis indicated that tyrosine, glutamic acid and lysine are frequently distributed around the acetylated lysine site, whereas isoleucine, phenylalanine and glutamic acid are frequently distributed around the succinylated lysine site in bermudagrass stolons (Fig. 3). Similar amino acid compositions were also found in other large-scale acetylation and succinylation identification studies. For example, tyrosine and lysine residues were frequently detected surrounding acetylated lysine sites in *P. asperata* [30], whereas glutamic acid was frequently detected surrounding succinylated lysine sites in both *C. cathayensis* and *D. officinale* [24, 27]. These results collectively imply that a conserved mechanism might exist in different plant species to add or/and remove acetylation and succinylation modifications to specific proteins. Considering that protein acyltransferases and succinyl transferases are still unidentified in plants, the biased non-enzymatic modification on lysine residues surrounded by specific amino acids might be a possible mechanism.

Acetylation, succinylation, as well as phosphorylation, are important PTMs that widely exist in proteins of all living organisms, especially enzymes involved in diverse metabolic reactions [52]. In fast-growing bermudagrass stolons, many enzymes catalyzing specific reactions in carbohydrate metabolism were also modified by acetylation, succinylation, and phosphorylation (Fig. 5). However, different enzymes participating in different metabolic pathways were preferentially modified by the three PTMs. Specifically, enzymes involved in the citrate cycle were highly succinylated, enzymes involved in starch and sucrose metabolism were highly phosphorylated, whereas enzymes involved in glycolysis were highly acetylated (Fig. 5). Notably, PK, which catalyzes the final rate-limiting step of glycolysis, was only acetylated, while AGPase catalyzing the rate-limiting step of starch synthesis was only phosphorylated [49]. One possible explanation for this phenomenon is feedback regulation theory [53]. Acetylation, succinylation and phosphorylation require acetyl-CoA, succinyl-CoA and ATP molecules, respectively. Through reversible acetylation, succinylation and phosphorylation, the activities of metabolic enzymes are strictly regulated to maintain the equilibrium of substrates and products, including the intermediate acetyl-CoA, succinyl-CoA and ATP molecules. As a link between glycolysis and the citrate cycle, PK catalyzes the transformation of phosphoenolpyruvate to pyruvate, which enters the mitochondrion to generate acetyl-CoA [54]. By contrast, AGPase catalyzes the formation of ADP-glucose by consuming ATP molecules [55]. Thus, acetylation of PK and phosphorylation of AGPase are both effective feedback regulation mechanisms to maintain the homeostasis of acetyl-CoA and ATP, respectively. Another possible explanation is non-enzymatic occurrence of acetylation and succinylation modifications [3, 5]. According to this theory, every solvent accessible lysine residue of proteins might be acetylated or succinylated, and stoichiometries of the two modifications are correlated with the concentration of acetyl-CoA and succinyl-CoA, respectively. The enrichment of acetylated proteins in glycolysis and succinylated proteins in the citrate cycle might result from relatively high concentrations of acetyl-CoA and succinyl-CoA molecules in the microenvironment of specific enzymes. In fast-growing plant organs, including bermudagrass stolons, the increase in cell numbers and growth of cells inevitably consume assimilated carbohydrates to synthesize proteins, nucleic acids and other biological macromolecules [56]. Through preferential modification of enzymes by different PTMs, diverse metabolic pathways, including starch degradation, glycolysis and the citrate cycle, are finely regulated to maintain the dynamic equilibrium of intermediate molecules, which in turn ensures efficient operation of the cell machinery during cell proliferation and expansion processes.

Although large-scale acetylome and succinylome studies have been successfully conducted in several plant species, the detailed regulatory functions of specific acetylation and succinylation modifications were only characterized in a few plants. For example, acetylation at K407 and K425 increased the enzyme activity of long-chain acyl-CoA synthetase in *P. tricornutum* [31]. Acetylation at three lysine sites all decreased the oxaloacetate reduction activity of the MDH protein in *A. thaliana*, while acetylation of the MDH protein at K172 increased its activity in *P. patens* [32]. Succinylation at K150 decreased the enzyme activity of a rice CAT protein, whereas succinylation at K55 or K241 increased the enzyme activity of a rice glutathione S‐transferase protein [10]. In this study, we expressed and purified wt and acetylation/deacetylation-mimic CAT proteins as well as wt and succinylation/desuccinylation-mimic MDH proteins. Enzymatic assays indicated that acetylation at K50 of CAT and succinylation at K332 of MDH both decreased their enzyme activities (Fig. 7). Considering that these results were obtained from in vitro purified proteins, the decrease of enzymatic activity of both acetylation-mimic CAT protein and succinylation-mimic MDH protein might only result from the change of protein charge, whereas deacetylation/desuccinylation-mimic mutations have little influence on the enzymatic activity because the charge of two proteins remain unchanged. Interestingly, both modification sites were conserved in the fast-growing organs of the three grasses. Previous studies have revealed that *Arabidopsis* mutant plants lacking mitochondrial MDH proteins exhibited small plant, slow growth and impaired seed post-germination growth phenotypes [57], while *Arabidopsis* catalase triple mutants also displayed severe redox disturbance and growth defects, implying that the two enzymes play important roles in fast organ growth [58]. The results of the current study further revealed that the activities of the two enzymes could be modulated by conserved acetylation and succinylation, respectively, providing new insights into the dynamic metabolic regulation in plant growth and development.

Our analyses successfully identified thousands of lysine acetylation modifications and hundreds of lysine succinylation modifications in fast-growing stolons of bermudagrass, however, many acetylated proteins and succinylated proteins possibly having important functions might not be determined due to their low abundance or poor reactivity with the affinity antibodies [43]. On the other hand, many studies have revealed that both acetylation and succinylation modifications exist in very low stoichiometries [5, 59–61]. Moreover, experimental evidence further indicated that ribulose-1,5-bisphosphate carboxylase/oxygenase (Rubisco) was acetylated at very low stoichiometries in *Arabidopsis* that could not meaningfully influence its enzymatic activity in vivo, and moderate increases in acetylation level didn’t significantly alter its maximal activity [62]. Considering that the purified acetylation/deacetylation-mimic CAT proteins and succinylation/desuccinylation-mimic MDH proteins could only simulate the 100% and 0% stoichiometry of acetylation and succinylation modifications on the two proteins, actual influences of the two modifications on the enzymatic activities in vivo might be weaker than that obtained from in vitro enzymatic assay results. In the future, we should not only use the state-of-the-art mass-spectrometry technology and high affinity antibodies to further explore the unknown acetylation and succinylation modifications but also determine the stoichiometries of interested modifications using isotope-labeled peptide standards to acquire their actual biological functions.

## Conclusion

In summary, 1914 acetylated proteins with 4657 lysine acetylation sites and 128 succinylated proteins with 226 lysine succinylation sites were first identified in bermudagrass stolons. The acetylated and succinylated proteins are located in different cell compartments, form complex interaction networks and participate in diverse cellular processes. The conserved acetylation of catalase and succinylation of malate dehydrogenase were further revealed to decrease enzyme activities in vitro, providing preliminary evidence of acetylation- and succinylation-mediated regulation of bermudagrass stolon growth at the biochemical level. In the future, with the available genome resources and genetic transformation techniques, the detailed physiological functions of specific acetylation and succinylation modifications could be elucidated in bermudagrass plants.

## Materials and methods

### Plant materials

The bermudagrass (*C. dactylon*) cultivar Yangjiang was used in this study. The bermudagrass plant was kindly provided by the National Warm-season Turfgrass Germplasm Resource Center of China and was grown in turfgrass plots of Yangzhou University (longitude and latitude: 32°35’ N, 119°40’ E; average annual temperatures: 22.4 °C; average annual precipitation: 1106 mm; annual average sunshine hours: 1960 h; soil type: 80% river sand and 20% peat soil) with standard turf management approaches.

### Inhibitor treatment and growth parameter measurements

The third stolon nodes were cut from bermudagrass plants, fixed in a floating foam board, transferred to Hoagland’s nutrient solution without (CK) and with different concentrations of sodium butyrate or sodium malonate, and grown in a growth chamber at 28 °C with a photoperiod of 16 h/8 h of white light (8000 lx) for two weeks. For each treatment, three plants regenerated from different stolon nodes were used as three biological replicates. The lengths of stolons and stolon internodes were measured using a ruler. Soluble sugar and protein contents were determined using the anthrone test and Bradford method, respectively [37]. Analysis of variance (ANOVA) with Tukey's multiple comparison test was used to determine the significant differences between CK and treatments.

### Total protein extraction, trypsin digestion and immunoaffinity enrichment of acetylated and succinylated peptides

Total proteins were extracted from the stolons of the bermudagrass plants using the Plant Total Protein Extraction Kit (Sangon Biotech, Shanghai, China) according to the manufacturer’s instructions. The protein concentration was also quantified using the Bradford method. For trypsin digestion, 100 μg of protein was firstly reduced with 10 mM DTT for 45 min at 50 °C and alkylated with 50 mM iodoacetamide for 45 min at room temperature in the dark. The protein solution was then diluted with four volumes of digestion buffer (100 mM TEAB, pH 8.0) containing 2 μg of Trypsin Gold (Promega, Madison, USA) and incubated at 37 °C overnight. The digested peptides were desalted using a Strata-X C18 SPE column (Phenomenex, Torrance, USA) and lyophilized by vacuum centrifugation. Acetylated and succinylated peptides were enriched from the lyophilized peptides using the PTMScan® Acetyl-Lysine Motif [Ac-K] Kit (Cell Signaling Technology, Danvers, USA) and PTMScan® Succinyl-Lysine Motif [Succ-K] Kit (Cell Signaling Technology) according to the manufacturer’s instructions, respectively.

### Liquid chromatography-tandem mass spectrometry (LC–MS/MS) analyses

LC–MS/MS analyses were performed using an EASY-nLC™ 1200 system (Thermo Fisher Scientific, Carlsbad, USA) coupled with a Q Exactive™ HF-X Hybrid Quadrupole-Orbitrap™ Mass Spectrometer (Thermo Fisher Scientific). Briefly, the enriched peptides were dissolved in solvent A (0.1% formic acid in 99.9% H_2_O), injected into a home-made C18 Nano-trap column (200 mm × 75 μm, 3 μm), and separated using a homemade reversed-phase analytical column (150 mm × 75 μm, 2 μm) through the EASY-nLC™ 1200 system. The gradient for LC separation was set as follows: starting from 8 to 16% solvent B (0.1% formic acid in 80% acetonitrile, 19.9% H_2_O) in 30 min, 16% to 35% solvent B in 50 min, and 35% to 45% solvent B in 35 min, thereafter increasing to 95% solvent B in 1 min and holding at 95% solvent B for the last 4 min, with a flow rate of 300 nL/min. The separated peptides were analyzed by the Q Exactive™ HF-X Hybrid Quadrupole-Orbitrap™ Mass Spectrometer with an ion source of Nanospray Flex™, spray voltage of 2.3 kV and ion transport capillary temperature of 320 °C in a data-dependent acquisition mode. The mass spectrometer was operated in a full scan manner that peptide ions in the mass range of 350–1500 m/z were all collected in a 0.08 s time window and the 30 most abundant ions were selected for automated MS/MS analysis with default parameters.

### Database searching and protein identification

The resulting MS/MS spectra were searched against the *C. dactylon* protein database (76,879 sequences, 33,188,769 residues) annotated from the genome using the Proteome Discoverer 2.2 software (Thermo Fisher Scientific) integrated with MaxQuant v1.6.7.0. The searching parameters were set as follows: cleavage enzyme, trypsin; maximum missing cleavages, 2; precursor ion mass tolerance, 10 ppm; fragment ion mass tolerance, 0.02 Da; fixed modification, carbamidomethylation of cystine (C); dynamic modifications, oxidation of methionine (M), acetylation or succinylation of lysine (K); minimum peptide length, 7; false discovery rate (FDR) threshold, 0.01; site localization probability, 0.75. Spectra reproducibly identified in two replicates were considered valid identification and used for further analysis.

### Bioinformatics analyses of acetylated and succinylated proteins

Annotation information of the identified acetylated and succinylated proteins was extracted from the bermudagrass genome annotation dataset [34]. Subcellular locations of acetylated and succinylated proteins were predicted using the Plant-mPLoc server (http://www.csbio.sjtu.edu.cn/bioinf/plant-multi/) [63]. For motif analysis, acetylated and succinylated peptides were extended to 15 amino acids in length with a modified lysine residue in the center and searched against the mis-cleaved peptides detected from the same proteins using the motif-x algorithm (https://meme-suite.org/meme/tools/momo) [64]. The minimum number of motif occurrences was set to 20 and the significance was set as *p* < 1 × 10^−6^. The occurrence ratio of seven upstream and downstream amino acids surrounding the acetylation or succinylation sites against their frequency in the MS identified total proteins was calculated, and a position-specific heatmap was generated by plotting the log_10_ of the ratios. KEGG enrichment analysis of acetylated and succinylated proteins was performed using a GO tool (https://agotool.org/) with MS identified total proteins as background and significance set at an FDR-corrected *p* value < 0.05 [65]. PPI information on acetylated and succinylated proteins was retrieved by searching the STRING v11 database (https://string-db.org/) with a high confidence score (> 0.7) and visualized by Cytoscape v3.9.1 [66, 67]. To identify orthologs of acetylated and succinylated proteins in other grass species, the amino acid sequence of each protein was BLAST searched against local protein databases comprising previously identified acetylated and succinylated proteins with an E-value threshold < 1 × 10^−6^.

### Gene cloning and site-specific mutation

Total RNA was extracted from the stolons of the bermudagrass plants using the RNAprep pure Plant kit (TIANGEN, Beijing, China). cDNA was synthesized using the SuperScript™ II reverse transcriptase kit (Takara, Dalian, China). The coding sequence (CDS) of catalase (Cd8B1G029850.1) and malate dehydrogenase (Cd2A1G010870.1) was PCR amplified from the cDNA and cloned into the pMD™19-T vector (Takara). Site-specific mutations of the two genes were generated using overlap PCR method with sequencing-confirmed clones as templates [68]. The used primers are listed in Table S11.

### Protein expression and purification

The CDS of wild-type (wt)/mutated catalase and malate dehydrogenase genes were PCR amplified, digested with restriction enzymes *Eco*R I/*Not* I and *Bam*H I/*Sal* I, and cloned into the prokaryotic expression vector pET-28a (Novagen, Wisconsin, USA). The recombinant His-tagged catalase and malate dehydrogenase proteins were expressed in *E. coli* BL21 (DE3) and purified using a nickel sulfate resin (Qiagen, Hilden, Germany) following the manufacturer’s instructions. The purity of the purification products was monitored through SDS-PAGE. Briefly, 40 μg of purified proteins was mixed with 6 × SDS-PAGE sampling buffer, denatured by boiling at 100 °C for 5 min, separated by 12% SDS-PAGE, and stained with Coomassie brilliant blue.

### Enzyme activity assay

Catalase activity was assayed by monitoring the elimination of H_2_O_2_ at 240 nm. The assay solution was composed of 100 mM K_2_HPO4-KH_2_PO4 (pH 7.0), 2.5 mM ferriheme chloride, 5 mM H_2_O_2_, and 1 μg of purified protein in a total volume of 1 ml [69]. Malate dehydrogenase activity was assayed by monitoring the reduction of NAD at 340 nm. The assay solution was composed of 120 mM glycine–NaOH (pH 10.0), 28 mM malate, 1.3 mM NAD and 1 μg of purified protein in a total volume of 1 ml [70]. All reactions were assayed for five technical replicates at 25 °C using an Ultrospec 3300 Pro spectrophotometer (Amersham Biosciences, Uppsala, Sweden) with a continuous recording function. The Vmax values were automatically calculated by the spectrophotometer. ANOVA with Tukey's multiple comparison test was used to determine the significant differences between different protein samples.

### Western blot detection of protein acetylation and succinylation

For western blotting, 20 μg of total proteins extracted from the stolons of different bermudagrass plants under different inhibitor treatments were mixed with 6 × SDS-PAGE sampling buffer, denatured by boiling at 100 °C for 5 min, separated by 12% SDS-PAGE and transferred to PVDF membranes. After blocking with nonfat skim milk, the PVDF membranes were incubated with anti-acetyllysine antibody (Order No.: PTM-105; PTM Biolabs, Hangzhou, China) and anti-succinyllysine antibody (Order No.: PTM-401; PTM Biolabs), respectively; followed by incubation with goat anti-rabbit conjugated to horseradish peroxidase (Abcam, Shanghai, China), and visualized with enhanced chemiluminescent substrate (Roche, Mannheim, Germany).

## Supplementary Information

**Additional file 1: Figure S1:** Western blot detection of holistic protein acetylation and succinylation levels in the stolons of bermudagrass plants under sodium butyrate and sodium malonate treatments. Protein (**A**) acetylation and (**B**) succinylation levels were decreased as the treatment concentrations of sodium butyrate and sodium malonate increase. **C** 20 μg of protein samples were run in SDSPAGE as a loading control for western blotting.

**Additional file 2: Figure S2:** Statistics of the matched spectra in acetylome and succinylome profiling data. Distribution of identified peptides according to the peptide length (**A**, **B**), precursor ion tolerance (**C**, **D**), unique peptide number (**E**, **F**) and protein coverage (**G**, **H**) in the acetylome (red color) and succinylome (green color) profiling experiments.

**Additional file 3: Figure S3:** Significant motifs identified by motif-X algorithm. Significant (**A**) acetylation and (**B**) succinylation motifs were represented using Sequence Log.

**Additional file 4: Figure S4:** Acetylation of ribosome subunit proteins in bermudagrass stolons. Proteins with/without PTMs were shown in red/black colors. The numbers of acetylation modification on each protein were marked as indicated.

**Additional file 5: Figure S5:** COG functional classification of the acetylated and succinylated proteins with conserved acetylation and succinylation site.

**Additional file 6: Figure S6:** Histone acetylation and succinylation identified in bermudagrass stolons. The 108 histone proteins were clustered according to their phylogenetic relationships. Histone proteins identified with acetylation and succinylation sites were marked with red and green circle, respectively. The four histone proteins sharing conserved acetylation sites with other grasses were marked using red font.

**Additional file 7: Figure S7:** Raw SDS-PAGE gel image showing the protein purification results. The cropped gels shown as figure 7C and D were extracted from this raw gel.

**Additional file 8: Table S1:** Statistics of peptide spectra obtained in this study.

**Additional file 9: Table S2:** Acetylated proteins and acetylated peptides identified in this study.

**Additional file 10: Table S3:** Succinylated proteins and succinylated peptides identified in this study.

**Additional file 11: Table S4:** Proteins with both acetylation and succinylation modifications.

**Additional file 12: Table S5:** KOBAS enrichment analysis of the acetylated proteins.

**Additional file 13: Table S6:** KOBAS enrichment analysis of the succinylated proteins.

**Additional file 14: Table S7:** Phosphoproteins and phosphopeptides previously identified in fast growing bermudagrass stolons.

**Additional file 15: Table S8:** Conserved acetylated proteins and acetylated sites in three grasses.

**Additional file 16: Table S9:** Conserved succinylated proteins and succinylated sites in three grasses.

**Additional file 17: Table S10:** Acetylation and succinylation modification of Histone proteins in bermudagrass stolon.

**Additional file 18: Table S11:** Primers used in this study.

## Data Availability

The acetylome and succinylome profiling mass spectrometry raw data have been deposited to the ProteomeXchange Consortium via the PRIDE partner repository with the dataset identifier PXD034841 (http://central.proteomexchange.org/PXD034841) and PXD034808 (http://central.proteomexchange.org/PXD034808), respectively.

## Abbreviations

ANOVA: Analysis of variance
CDS: Coding sequence
COG: Clusters of Orthologous Genes
KEGG: Kyoto Encyclopedia of Genes and Genomes
LC–MS/MS: Liquid chromatography coupled to tandem mass spectrometry
PPI: Protein–protein interaction
PTM: Post-translational modifications
wt: Wild-type
SPP: Sucrose-phosphatase
INV: Invertase
SuS: Sucrose synthase
SPS: Sucrose-phosphate synthase
UGP: UTP--glucose-1-phosphate uridylyltransferase
PGM: Phosphoglucomutase
HXK: Hexokinase
GPI: Glucose-6-phosphate isomerase
PFP: Pyrophosphate--fructose 6-phosphate 1-phosphotransferase
FBP: Fructose-1,6-bisphosphatase
FBA: Fructose-bisphosphate aldolase
TPI: Triosephosphate isomerase
GAPDH: Glyceraldehyde-3-phosphate dehydrogenase
PGK: Phosphoglycerate kinase
PGAM: Phosphoglycerate mutase
ENO: Enolase
PK: Pyruvate kinase
PEPC: Phosphoenolpyruvate carboxylase
MDH: Malate dehydrogenase
PDH-E1: Pyruvate dehydrogenase
PDH-E2: Dihydrolipoamide acetyltransferase
PDH/ODH-E3: Dihydrolipoyl dehydrogenase
ME: Malic enzyme
CS: Citrate synthase
AH: Aconitate hydratase
IDH: Isocitrate dehydrogenase
ODH-E1: Oxoglutarate dehydrogenase (succinyl-transferring)
ODH-E2: Dihydrolipoyllysine-residue succinyltransferase
SUC: Succinate--CoA ligase
SDH: Succinate dehydrogenase
FH: Fumarate hydratase
AMY: Amylase
ISA: Isoamylase
PULA: Pullulanase
SBE: 1,4-Alpha-glucan-branching enzyme
SS: Starch synthase
SP: Starch phosphorylase
AGPase: ADP-glucose pyrophosphorylase
TKL: Transketolase
TAL: Transaldolase
SBP: Sedoheptulose-1,7-bisphosphatase.
RPI: Ribose-5-phosphate isomerase
RPE: Ribulose-5-phosphate epimerase
PRK: Ribulose-5-phosphate kinase
Rubisco: Ribulose bisphosphate carboxylase/oxygenase
