# Supplementary material for: Proteome-wide analyses reveal diverse functions of protein acetylation and succinylation modifications in fast growing stolons of bermudagrass (Cynodon dactylon L.)
Source: BMC Plant Biol. 2022 Oct 27;22:503. doi: 10.1186/s12870-022-03885-2 (PMC9608919; doi:10.1186/s12870-022-03885-2)
Supplement: Supplementary file 1 — Additional file 1: Figure S1: Western blot detection of holistic protein acetylation and succinylation levels in the stolons of bermudagrass plants under sodium butyrate and sodium malonate treatments. Protein (A) acetylation and (B) succinylation levels were decreased as the treatment concentrations of sodium butyrate and sodium malonate increase. C 20 μg of protein samples were run in SDSPAGE as a loading control for western blotting. [file 12870_2022_3885_MOESM1_ESM.pdf]

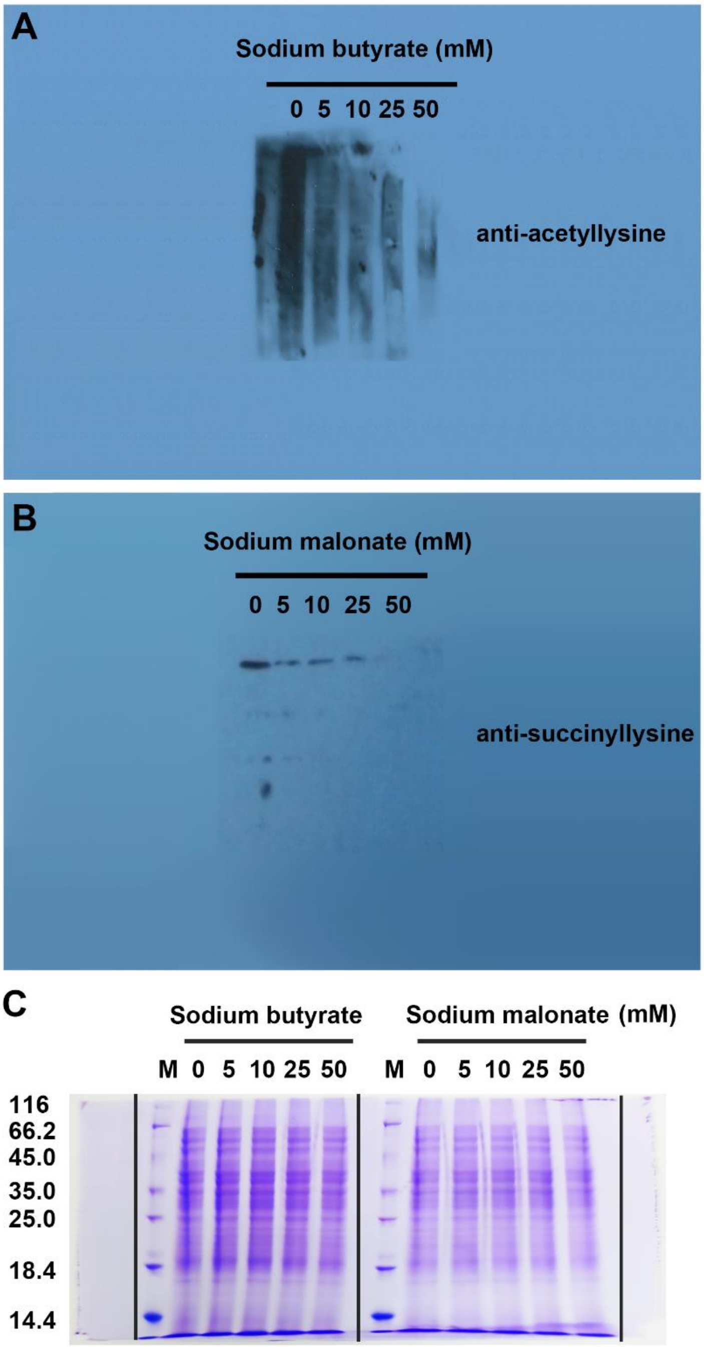

Figure S1. Western blot detection of holistic protein acetylation and succinylation levels in the stolons of bermudagrass plants under sodium butyrate and sodium malonate treatments

Protein **(A)** acetylation and **(B)** succinylation levels were decreased as the treatment concentrations of sodium butyrate and sodium malonate increase. **(C)** 20 μg of protein samples were run in SDS-PAGE as a loading control for western blotting.
