# Supplementary material for: Proteome-wide analyses reveal diverse functions of protein acetylation and succinylation modifications in fast growing stolons of bermudagrass (Cynodon dactylon L.)
Source: BMC Plant Biol. 2022 Oct 27;22:503. doi: 10.1186/s12870-022-03885-2 (PMC9608919; doi:10.1186/s12870-022-03885-2)
Supplement: Supplementary file 2 — Additional file 2: Figure S2: Statistics of the matched spectra in acetylome and succinylome profiling data. Distribution of identified peptides according to the peptide length (A, B), precursor ion tolerance (C, D), unique peptide number (E, F) and protein coverage (G, H) in the acetylome (red color) and succinylome (green color) profiling experiments. [file 12870_2022_3885_MOESM2_ESM.pdf]

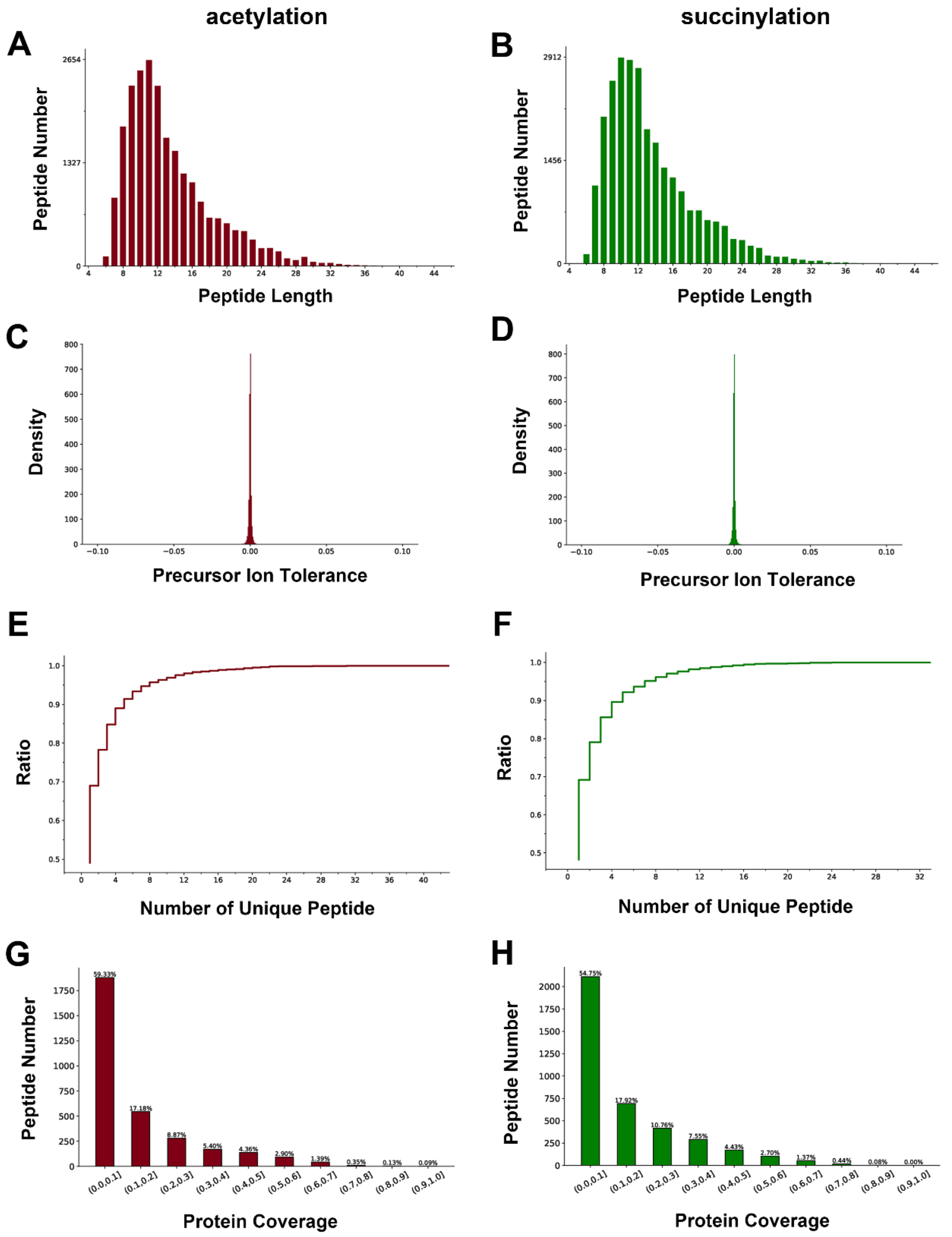

Figure S2. Statistics of the matched spectra in acetylome and succinylome profiling data

Distribution of identified peptides according to the peptide length (**A**, **B**), precursor ion tolerance (**C**, **D**), unique peptide number (**E**, **F**) and protein coverage (**G**, **H**) in the acetylome (red color) and succinylome (green color) profiling experiments.
