# Supplementary material for: Proteome-wide analyses reveal diverse functions of protein acetylation and succinylation modifications in fast growing stolons of bermudagrass (Cynodon dactylon L.)
Source: BMC Plant Biol. 2022 Oct 27;22:503. doi: 10.1186/s12870-022-03885-2 (PMC9608919; doi:10.1186/s12870-022-03885-2)
Supplement: Supplementary file 3 — Additional file 3: Figure S3: Significant motifs identified by motif-X algorithm. Significant (A) acetylation and (B) succinylation motifs were represented using Sequence Log. [file 12870_2022_3885_MOESM3_ESM.pdf]

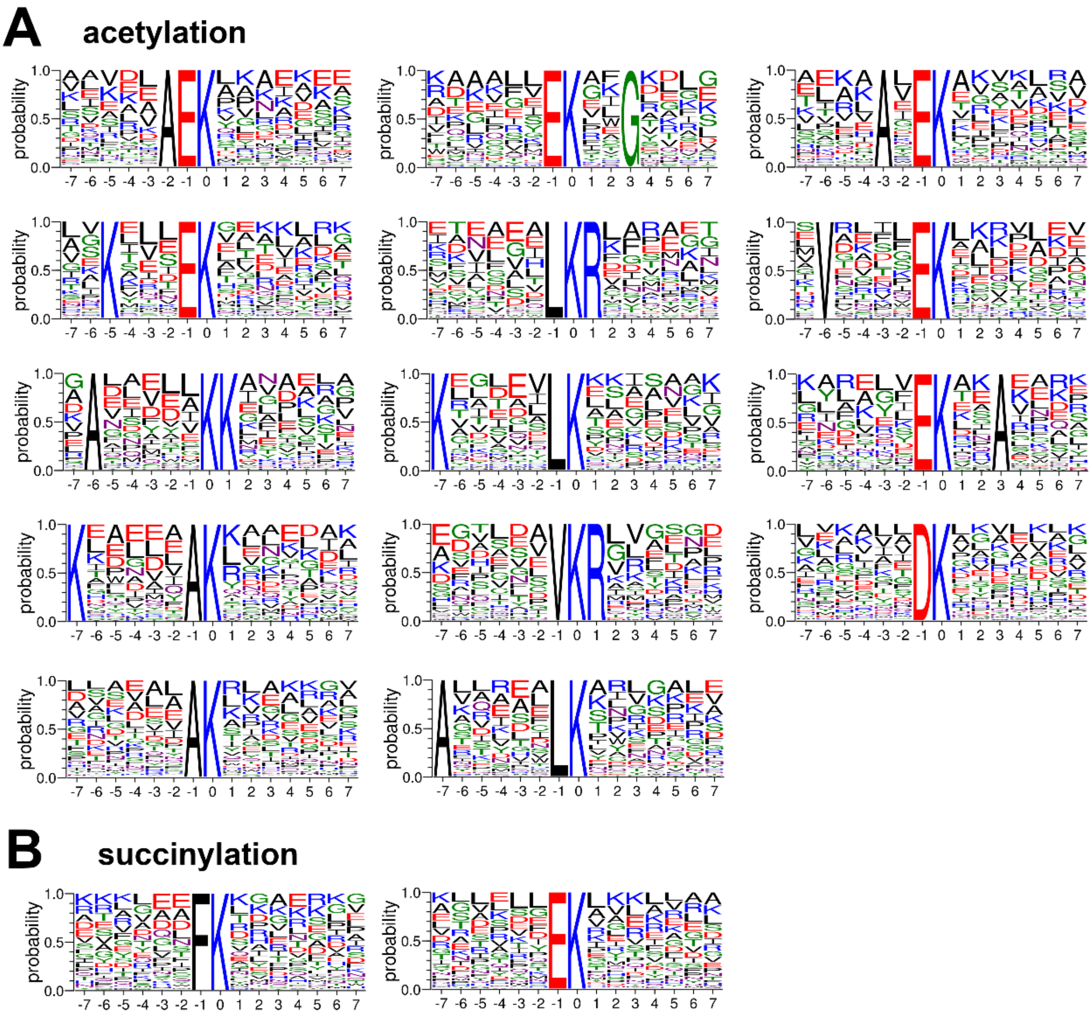

Figure S3. Significant motifs identified by motif-X algorithm

Significant **(A)** acetylation and **(B)** succinylation motifs were represented using Sequence Logo
