# Supplementary material for: Proteome-wide analyses reveal diverse functions of protein acetylation and succinylation modifications in fast growing stolons of bermudagrass (Cynodon dactylon L.)
Source: BMC Plant Biol. 2022 Oct 27;22:503. doi: 10.1186/s12870-022-03885-2 (PMC9608919; doi:10.1186/s12870-022-03885-2)
Supplement: Supplementary file 4 — Additional file 4: Figure S4: Acetylation of ribosome subunit proteins in bermudagrass stolons. Proteins with/without PTMs were shown in red/black colors. The numbers of acetylation modification on each protein were marked as indicated. [file 12870_2022_3885_MOESM4_ESM.pdf]

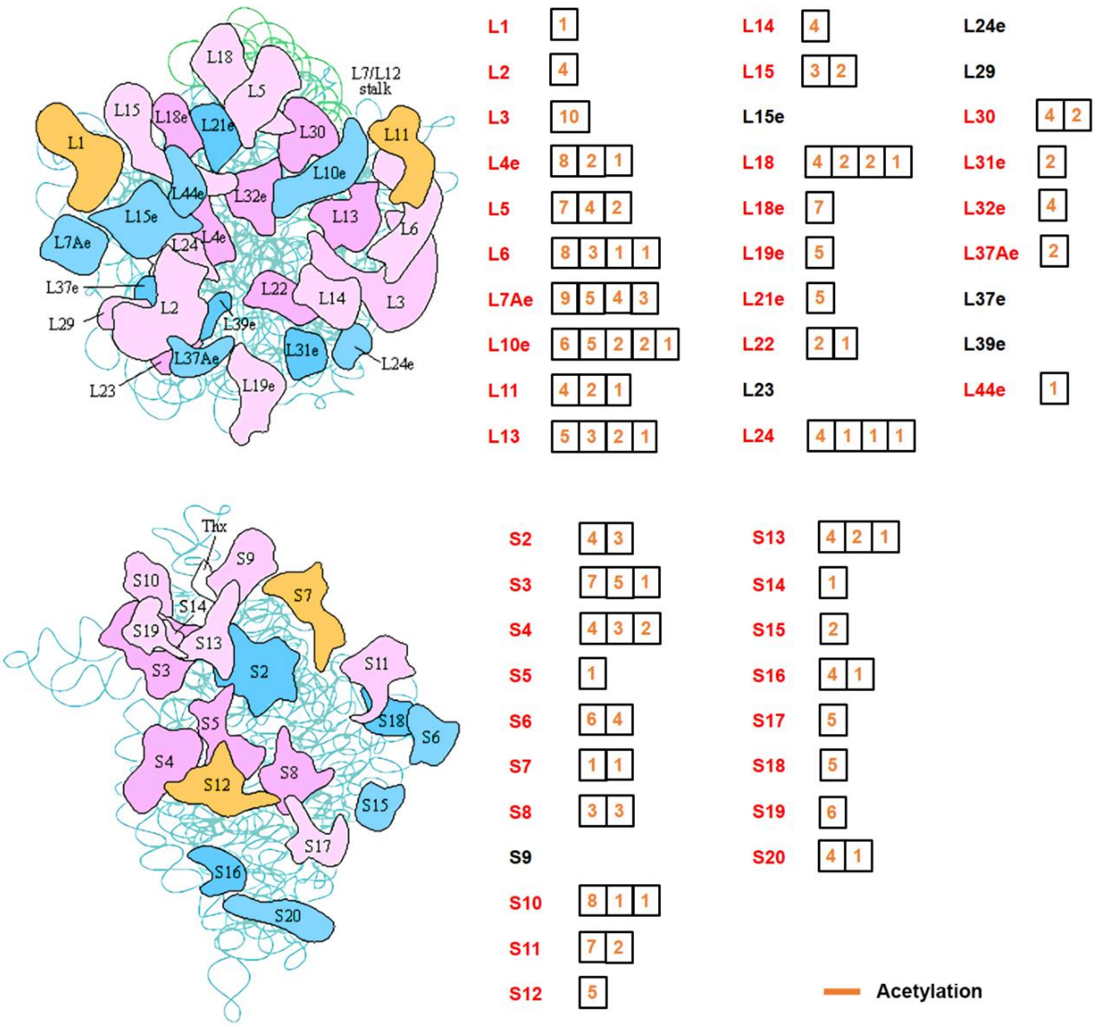

Figure S4. Acetylation of ribosome subunit proteins in bermudagrass stolons

Proteins with/without PTMs were shown in red/black colors. The numbers of acetylation modification on each protein were marked as indicated.
