# Supplementary material for: Proteome-wide analyses reveal diverse functions of protein acetylation and succinylation modifications in fast growing stolons of bermudagrass (Cynodon dactylon L.)
Source: BMC Plant Biol. 2022 Oct 27;22:503. doi: 10.1186/s12870-022-03885-2 (PMC9608919; doi:10.1186/s12870-022-03885-2)
Supplement: Supplementary file 5 — Additional file 5: Figure S5: COG functional classification of the acetylated and succinylated proteins with conserved acetylation and succinylation site. [file 12870_2022_3885_MOESM5_ESM.pdf]

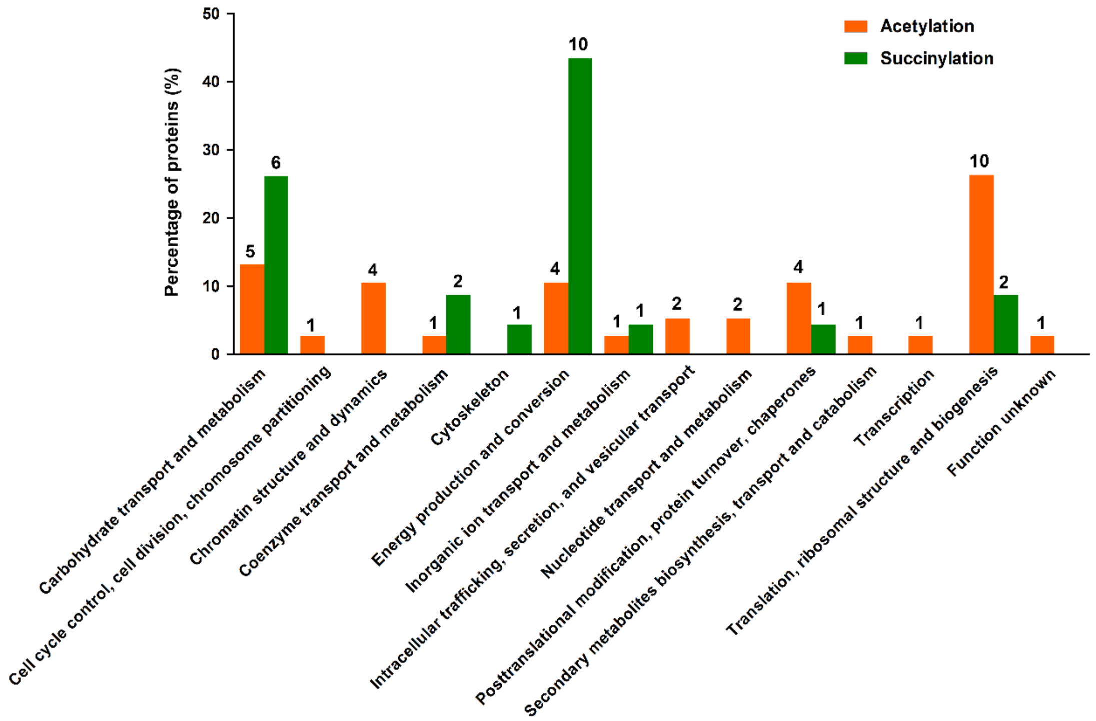

Figure S5. COG functional classification of the acetylated and succinylated proteins with conserved acetylation and succinylation sites
