# Supplementary material for: Proteome-wide analyses reveal diverse functions of protein acetylation and succinylation modifications in fast growing stolons of bermudagrass (Cynodon dactylon L.)
Source: BMC Plant Biol. 2022 Oct 27;22:503. doi: 10.1186/s12870-022-03885-2 (PMC9608919; doi:10.1186/s12870-022-03885-2)
Supplement: Supplementary file 6 — Additional file 6: Figure S6: Histone acetylation and succinylation identified in bermudagrass stolons. The 108 histone proteins were clustered according to their phylogenetic relationships. Histone proteins identified with acetylation and succinylation sites were marked with red and green circle, respectively. The four histone proteins sharing conserved acetylation sites with other grasses were marked using red font. [file 12870_2022_3885_MOESM6_ESM.pdf]

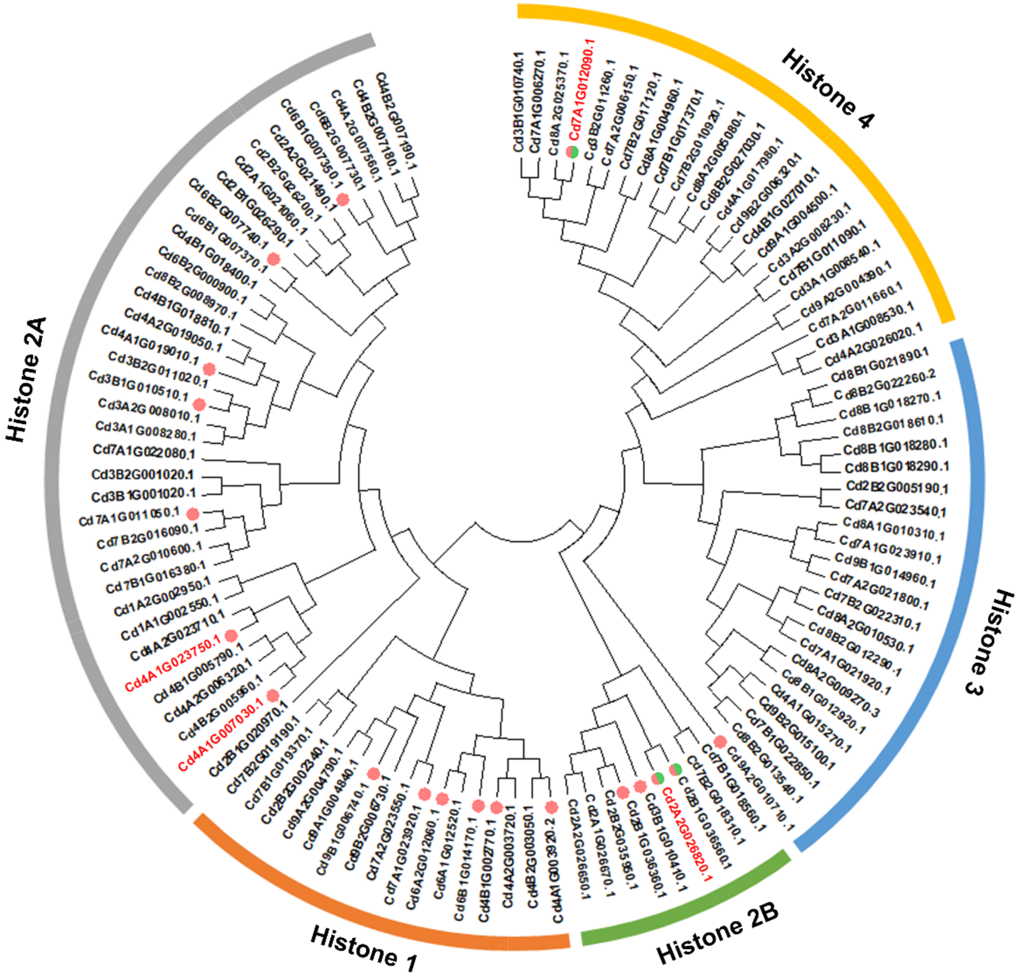

Figure S6. Histone acetylation and succinylation identified in bermudagrass stolons

The 108 histone proteins were clustered according to their phylogenetic relationships. Histone proteins identified with acetylation and succinylation sites were marked with red and green circle, respectively. The four histone proteins sharing conserved acetylation sites with other grasses were marked using red font.
