# Supplementary material for: Proteome-wide analyses reveal diverse functions of protein acetylation and succinylation modifications in fast growing stolons of bermudagrass (Cynodon dactylon L.)
Source: BMC Plant Biol. 2022 Oct 27;22:503. doi: 10.1186/s12870-022-03885-2 (PMC9608919; doi:10.1186/s12870-022-03885-2)
Supplement: Supplementary file 7 — Additional file 7: Figure S7: Raw SDS-PAGE gel image showing the protein purification results. The cropped gels shown as figure 7C and D were extracted from this raw gel. [file 12870_2022_3885_MOESM7_ESM.pdf]

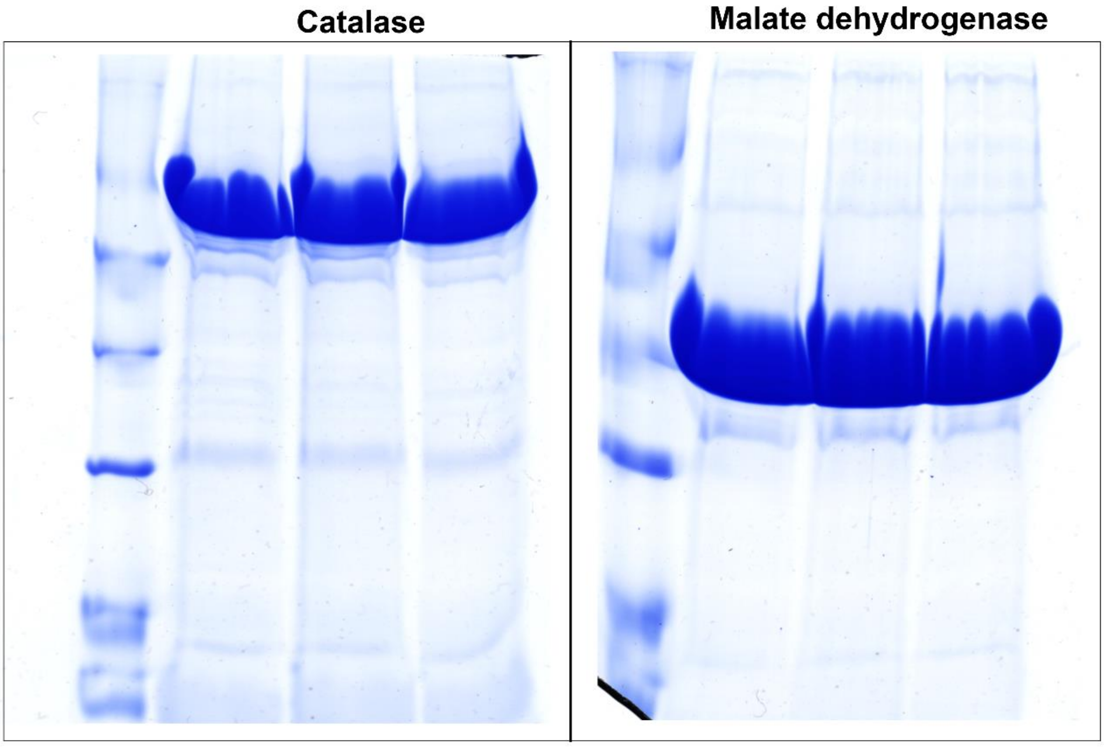

Figure S7. Raw SDS-PAGE gel image showing the protein purification results

The cropped gels shown as figure 7C and 7D were extracted from this raw gel.
