# Supplementary material for: Proteome-wide analyses reveal diverse functions of protein acetylation and succinylation modifications in fast growing stolons of bermudagrass (Cynodon dactylon L.)
Source: BMC Plant Biol. 2022 Oct 27;22:503. doi: 10.1186/s12870-022-03885-2 (PMC9608919; doi:10.1186/s12870-022-03885-2)
Supplement: Supplementary file 8 — Additional file 8: Table S1: Statistics of peptide spectra obtained in this study. [file 12870_2022_3885_MOESM8_ESM.pdf]

Table S1. Statistics of peptide spectra obtained in this study

Experiments	Total spectra	Matched spectra	Modified peptide	Modified site	Modified protein
acetylation-R1	60849	24238	5247	4708	1962
acetylation-R2	62183	25482	5291	4816	1996
succinylation-R1	61215	24815	253	244	142
succinylation-R2	60752	24162	248	236	137
