# Supplementary material for: Proteome-wide analyses reveal diverse functions of protein acetylation and succinylation modifications in fast growing stolons of bermudagrass (Cynodon dactylon L.)
Source: BMC Plant Biol. 2022 Oct 27;22:503. doi: 10.1186/s12870-022-03885-2 (PMC9608919; doi:10.1186/s12870-022-03885-2)
Supplement: Supplementary file 12 — Additional file 12: Table S5: KOBAS enrichment analysis of the acetylated proteins. [file 12870_2022_3885_MOESM12_ESM.pdf]

Table S5. KOBAS enrichment analysis of the acetylated proteins

KEGG pathway	Description	Observed proteins	Background proteins	*p*-value
KO03010	Ribosome	98	121	0.00000252
KO00010	Glycolysis / Gluconeogenesis	55	85	0.00041400
KO03050	Proteasome	27	42	0.00431000
KO00020	Citrate cycle (TCA cycle)	28	41	0.06910000
KO00730	Thiamine metabolism	7	16	0.02098402
KO00190	Oxidative phosphorylation	43	61	0.03359226
KO01230	Biosynthesis of amino acids	93	144	0.04741279
KO00480	Glutathione metabolism	41	60	0.07847685
KO01200	Carbon metabolism	110	175	0.08218003
KO00300	Lysine biosynthesis	8	9	0.08644570
KO00195	Photosynthesis	22	30	0.08994703
KO00710	Carbon fixation in photosynthetic organisms	43	64	0.10114695
KO00330	Arginine and proline metabolism	14	18	0.11076235
KO00640	Propanoate metabolism	14	18	0.11076235
KO00270	Cysteine and methionine metabolism	31	45	0.12031983
KO00620	Pyruvate metabolism	40	60	0.13535722
KO00360	Phenylalanine metabolism	15	20	0.14563888
KO00280	Valine, leucine and isoleucine degradation	18	25	0.16900163
KO00941	Flavonoid biosynthesis	8	10	0.20117687
KO00040	Pentose and glucuronate interconversions	10	13	0.22540558
KO00590	Arachidonic acid metabolism	3	3	0.26208605
KO00790	Folate biosynthesis	3	3	0.26208605
KO03030	DNA replication	3	3	0.26208605
KO03430	Mismatch repair	3	3	0.26208605
KO00950	Isoquinoline alkaloid biosynthesis	7	9	0.31368181
KO00071	Fatty acid degradation	14	20	0.31694261
KO00960	Tropane, piperidine and pyridine alkaloid biosynthesis	8	11	0.36650748
KO03008	Ribosome biogenesis in eukaryotes	8	11	0.36650748
KO03050	Proteasome	27	42	0.38028271
KO03420	Nucleotide excision repair	4	5	0.39501073
KO00053	Ascorbate and aldarate metabolism	19	29	0.42247530
KO00410	beta-Alanine metabolism	14	21	0.46756391
KO00030	Pentose phosphate pathway	27	43	0.48962885
KO00400	Phenylalanine, tyrosine and tryptophan biosynthesis	19	30	0.56284928
KO00970	Aminoacyl-tRNA biosynthesis	19	30	0.56284928
KO00072	Synthesis and degradation of ketone bodies	3	4	0.63700644
KO00750	Vitamin B6 metabolism	3	4	0.63700644
KO04712	Circadian rhythm - plant	3	4	0.63700644
KO00220	Arginine biosynthesis	14	22	0.64184276
KO04016	KOK signaling pathway - plant	11	17	0.65872428
KO01212	Fatty acid metabolism	15	24	0.69523378
KO00261	Monobactam biosynthesis	5	7	0.70567702
KO00250	Alanine, aspartate and glutamate metabolism	23	38	0.73126343
KO00340	Histidine metabolism	6	9	0.73937236
KO00380	Tryptophan metabolism	6	9	0.73937236
KO00310	Lysine degradation	7	11	0.76494778
KO00910	Nitrogen metabolism	7	11	0.76494778
KO01210	2-Oxocarboxylic acid metabolism	18	30	0.83658755
KO00630	Glyoxylate and dicarboxylate metabolism	23	39	0.87757320
KO00260	Glycine, serine and threonine metabolism	19	32	0.87835848
KO00061	Fatty acid biosynthesis	9	15	0.98873242
KO00130	Ubiquinone and other terpenoid-quinone biosynthesis	8	14	1.00000000
KO00240	Pyrimidine metabolism	17	30	1.00000000
KO00290	Valine, leucine and isoleucine biosynthesis	4	7	1.00000000
KO00440	Phosphonate and phosphinate metabolism	1	1	1.00000000
KO00670	One carbon pool by folate	6	10	1.00000000
KO00909	Sesquiterpenoid and triterpenoid biosynthesis	1	1	1.00000000
KO00945	Stilbenoid, diarylheptanoid and gingerol biosynthesis	4	7	1.00000000
KO00966	Glucosinolate biosynthesis	1	1	1.00000000
KO03410	Base excision repair	1	1	1.00000000
