# Supplementary material for: Proteome-wide analyses reveal diverse functions of protein acetylation and succinylation modifications in fast growing stolons of bermudagrass (Cynodon dactylon L.)
Source: BMC Plant Biol. 2022 Oct 27;22:503. doi: 10.1186/s12870-022-03885-2 (PMC9608919; doi:10.1186/s12870-022-03885-2)
Supplement: Supplementary file 13 — Additional file 13: Table S6: KOBAS enrichment analysis of the succinylated proteins. [file 12870_2022_3885_MOESM13_ESM.pdf]

Table S6. KOBAS enrichment analysis of the succinylated proteins

KEGG pathway	Description	Observed proteins	Background proteins	*p*-value
KO00020	Citrate cycle (TCA cycle)	12	52	2.90E-08
KO00190	Oxidative phosphorylation	9	75	0.0009612
KO00310	Lysine degradation	3	16	0.0462765
KO00630	Glyoxylate and dicarboxylate metabolism	5	49	0.0462765
KO00640	Propanoate metabolism	3	17	0.0462765
KO00620	Pyruvate metabolism	6	73	0.0462765
KO00010	Glycolysis / Gluconeogenesis	7	97	0.0462765
KO00260	Glycine, serine and threonine metabolism	4	42	0.0809982
KO00670	One carbon pool by folate	2	9	0.0809982
KO01110	Biosynthesis of secondary metabolites	20	504	0.0815673
KO00280	Valine, leucine and isoleucine degradation	3	27	0.0986823
KO00710	Carbon fixation in photosynthetic organisms	5	72	0.1033108
KO03018	RNA degradation	3	33	0.1436569
KO04130	SNARE interactions in vesicular transport	1	5	0.3323781
KO00270	Cysteine and methionine metabolism	3	50	0.3395133
KO00195	Photosynthesis	2	31	0.4316909
KO00950	Isoquinoline alkaloid biosynthesis	1	8	0.4316909
KO01210	2-Oxocarboxylic acid metabolism	2	34	0.4684781
KO00960	Tropane, piperidine and pyridine alkaloid biosynthesis	1	10	0.4765304
KO04145	Phagosome	2	39	0.4826656
KO01230	Biosynthesis of amino acids	6	150	0.4826656
KO00250	Alanine, aspartate and glutamate metabolism	2	41	0.4826656
KO00380	Tryptophan metabolism	1	13	0.4826656
KO00650	Butanoate metabolism	1	14	0.4946195
KO00040	Pentose and glucuronate interconversions	1	15	0.5050004
KO00196	Photosynthesis - antenna proteins	1	16	0.513988
KO00350	Tyrosine metabolism	1	20	0.5338908
KO00052	Galactose metabolism	1	21	0.5338908
KO00330	Arginine and proline metabolism	1	21	0.5338908
KO00561	Glycerolipid metabolism	1	21	0.5338908
KO00220	Arginine biosynthesis	1	22	0.5338908
KO00360	Phenylalanine metabolism	1	22	0.5338908
KO00410	beta-Alanine metabolism	1	23	0.5338908
KO00562	Inositol phosphate metabolism	1	23	0.5338908
KO00400	Phenylalanine, tyrosine and tryptophan biosynthesis	1	25	0.5523127
KO04146	Peroxisome	1	31	0.6239324
KO00051	Fructose and mannose metabolism	1	39	1
