# Supplementary material for: Proteome-wide analyses reveal diverse functions of protein acetylation and succinylation modifications in fast growing stolons of bermudagrass (Cynodon dactylon L.)
Source: BMC Plant Biol. 2022 Oct 27;22:503. doi: 10.1186/s12870-022-03885-2 (PMC9608919; doi:10.1186/s12870-022-03885-2)
Supplement: Supplementary file 17 — Additional file 17: Table S10: Acetylation and succinylation modification of Histone proteins in bermudagrass stolon. [file 12870_2022_3885_MOESM17_ESM.pdf]

Table S10. Acetylation and succinylation modification of Histone proteins in bermudagrass stolons

Accession number in genome	Annotation	Acetylated peptides	Succinylated peptides
Cd4A1G003920.2	Histone H1	TGSSQYAIAKFVEDK<br>ANPTHPPYAEMISEAIASLKER<br>LVAAGKLAK<br>LLLVQLKK<br>AAPAKKPAAAAK	-
Cd4B1G002770.1	Histone H1	FVEDKQK<br>NSYKLPPTR<br>LVAAGKLTK<br>YVEEKHGANLSPNFR	-
Cd6A2G012060.1	Histone H1	TGSSSVAISKYVEEK<br>DSPAAKKPAAAAK	-
Cd6B1G014170.1	Histone H1	LLSGQLKK<br>YVEEKHGANLPPNFR<br>EKTGSSSVAISK	-
Cd7A1G023920.1	Histone H1	APAKKPVAAAPAPK	-
Cd9B1G006740.1	Histone H1	HGASLPANYKK	-
Cd4A1G023750.1	Histone H2A.1	GLLAAKTTAAKSTDK	-
Cd6B1G007350.1	Histone H2A.2	AIGSGTAKKAMSR<br>GKAIGSGTAKK	-
Cd4A1G007030.1	Histone H2A.3	TTAAKSAEKDK<br>GTIAGGGVIPHIHKSLINK<br>GGKGLLAAKTTAAK	-
Cd3B1G010510.1	Histone H2A.4	SVKAGLQFPVGR	-
Cd4A1G019010.1	Histone H2A.5	NDEELGKLLSGVTIAHGGVLPNINPVLLP<br>LLSGVTIAHGGVLPNINPVLLPKK<br>MDSTGTGAGGKVK	-
Cd7A1G011050.1	Histone H2A.5	SVKAGLQFPVSR	-
Cd6B2G007740.1	Histone H2A.7	AIGAGAAKKATSR<br>GKAIGAGAAKK	-
Cd2B1G036560.1	Histone H2B.4	KPAAKKPAGEEPATEK<br>LAGEAAKLAR<br>AEKKPAAKKPAGEEPATEK<br>KPAGEEPATEKAEK<br>AEKAPGGKKPK	LAGEAAKLAR
Cd2A2G026820.1	Histone H2B.5	LVLPGELAKHAVSEGTK<br>LAAEAAKLAR<br>KPAEEEPATEKAEKAPAGK<br>VLKQVHPDIGISSK<br>AEKKPAAKKPAEEEPATEK<br>IYIFKVLK<br>LPAGKSAGKEGGDK<br>SVETYKIYIFK<br>AEKAPAGKKPK	KPAEEEPATEK<br>LAAEAAKLAR<br>LVLPGELAKHAVSEGTK<br>VLKQVHPDIGISSK<br>YNKKPTITSR
Cd2B1G036360.1	Histone	RLPAGKSVGK	-
Cd3B1G010410.1	Histone H2B.11	KPAEKKPAEEK<br>AEKKPAEKKPAEEK	-
Cd9A2G010710.1	Histone H3.3	KSAPTTGGVKKPHR	-
Cd7A1G012090.1	Histone H4	TVTAMDVVYALKR<br>GLGKGGAKR<br>GGKGLGKGGAK	DNIQGITKPAIR
