# Supplementary material for: Proteome-wide analyses reveal diverse functions of protein acetylation and succinylation modifications in fast growing stolons of bermudagrass (Cynodon dactylon L.)
Source: BMC Plant Biol. 2022 Oct 27;22:503. doi: 10.1186/s12870-022-03885-2 (PMC9608919; doi:10.1186/s12870-022-03885-2)
Supplement: Supplementary file 18 — Additional file 18: Table S11: Primers used in this study. [file 12870_2022_3885_MOESM18_ESM.pdf]

Table S11. Primers used in this study

Name	Usage	Sequences
CAT-F	Gene cloning	ATGGATCCTACCAAGTTCCGC
CAT-R		TCACATGCTTGGCTTGACGTT
MDH-F		ATGAGGCCGTCGATGATGAGA
MDH-R		CTAGTTCTCATTTGCAAATTT
CAT-K50R-R	Site-specific mutation	GTGCGCAACCCTCTCGATCAGGTGGTAGTCC
CAT-K50R-F		CTGATCGAGAGGGTTGCGCACTTCGCCCGGG
CAT-K50Q-R		TGCGCAACCTGCTCGATCAGGTGGTAGTCCT
CAT-K50Q-F		CCTGATCGAGCAGGTTGCGCACTTCGCCCGG
MDH-K332R-R		TTTGATTCCCCTCTCAATGGAAGACTTGAGC
MDH-K332R-F		TCCATTGAGAGGGGAATCAAATTTGCAAATG
MDH-K332E-R		TTGATTCCCTCCTCAATGGAAGACTTGAGCT
MDH-K332E-F		TTCCATTGAGGAGGGAATCAAATTTGCAAAT
CAT-OE-F	Protein expression	CGGAATTCATGGATCCTACCAAGTTCCGC
CAT-OE-R		CGGCGGCCGCTCACATGCTTGGCTTGACGTT
MDH-OE-F		CGGGATCCATGAGGCCGTCGATGATGAGA
MDH-OE-R		CGGTCGACCTAGTTCTCATTTGCAAATTT

Note: the mutation sites and restriction endonuclease recognition sites were marked with underline
